# Real-World Implementation of Video Outpatient Consultations at Macro, Meso, and Micro Levels: Mixed-Method Study

**DOI:** 10.2196/jmir.9897

**Published:** 2018-04-17

**Authors:** Trisha Greenhalgh, Sara Shaw, Joseph Wherton, Shanti Vijayaraghavan, Joanne Morris, Satya Bhattacharya, Philippa Hanson, Desirée Campbell-Richards, Seendy Ramoutar, Anna Collard, Isabel Hodkinson

**Affiliations:** ^1^ Nuffield Department of Primary Care Health Sciences University of Oxford Oxford United Kingdom; ^2^ Barts Health NHS Trust London United Kingdom; ^3^ Tower Hamlets Clinical Commissioning Group London United Kingdom

**Keywords:** remote consultations, diabetes mellitus, ethnography, interviews, organizational case studies, health systems

## Abstract

**Background:**

There is much interest in virtual consultations using video technology. Randomized controlled trials have shown video consultations to be acceptable, safe, and effective in selected conditions and circumstances. However, this model has rarely been mainstreamed and sustained in real-world settings.

**Objective:**

The study sought to (1) define good practice and inform implementation of video outpatient consultations and (2) generate transferable knowledge about challenges to scaling up and routinizing this service model.

**Methods:**

A multilevel, mixed-method study of Skype video consultations (micro level) was embedded in an organizational case study (meso level), taking account of national context and wider influences (macro level). The study followed the introduction of video outpatient consultations in three clinical services (diabetes, diabetes antenatal, and cancer surgery) in a National Health Service trust (covering three hospitals) in London, United Kingdom. Data sources included 36 national-level stakeholders (exploratory and semistructured interviews), longitudinal organizational ethnography (300 hours of observations; 24 staff interviews), 30 videotaped remote consultations, 17 audiotaped face-to-face consultations, and national and local documents. Qualitative data, analyzed using sociotechnical change theories, addressed staff and patient experience and organizational and system drivers. Quantitative data, analyzed via descriptive statistics, included uptake of video consultations by staff and patients and microcategorization of different kinds of talk (using the Roter interaction analysis system).

**Results:**

When clinical, technical, and practical preconditions were met, video consultations appeared safe and were popular with some patients and staff. Compared with face-to-face consultations for similar conditions, video consultations were very slightly shorter, patients did slightly more talking, and both parties sometimes needed to make explicit things that typically remained implicit in a traditional encounter. Video consultations appeared to work better when the clinician and patient already knew and trusted each other. Some clinicians used Skype adaptively to respond to patient requests for ad hoc encounters in a way that appeared to strengthen supported self-management. The reality of establishing video outpatient services in a busy and financially stretched acute hospital setting proved more complex and time-consuming than originally anticipated. By the end of this study, between 2% and 22% of consultations were being undertaken remotely by participating clinicians. In the remainder, clinicians chose not to participate, or video consultations were considered impractical, technically unachievable, or clinically inadvisable. Technical challenges were typically minor but potentially prohibitive.

**Conclusions:**

Video outpatient consultations appear safe, effective, and convenient for patients in situations where participating clinicians judge them clinically appropriate, but such situations are a fraction of the overall clinic workload. As with other technological innovations, some clinicians will adopt readily, whereas others will need incentives and support. There are complex challenges to embedding video consultation services within routine practice in organizations that are hesitant to change, especially in times of austerity.

## Introduction

### Background

Outpatient services, particularly for people with long-term conditions, have changed little in recent decades. Yet, in many countries, population demographics, disease epidemiology, and care priorities have changed a great deal, with, for example, an aging population, rising rates of chronic illness and multimorbidity, and an increasing emphasis on multidisciplinary team care and supported self-management. Outpatient nonattendance rates in some patient groups, especially the disadvantaged and those with multiple and complex needs, are high and may be associated with poor disease control, increased use of emergency services, and high costs [1,2]. Patient-borne costs (in terms of time and travel) of attending outpatient appointments are high, especially for tertiary care [3-5].

There is a strong policy push in the United Kingdom [6-9] and elsewhere [10-12] to harness the potential of digital technologies to improve care models and redesign care pathways in a way that improves the accessibility and efficiency of services and maximizes the potential for patient self-management. The UK’s National Information Board recently argued that a different kind of health service is needed, in which the traditional outpatient consultation will become increasingly obsolete [8]. Remote video consulting using Skype or FaceTime is one potential solution.

Published research on video outpatient consultations has been summarized in several recent narrative reviews [13-15]. Randomized controlled trials (RCTs) have shown such consultations to be acceptable, safe, and effective (and, when measured, to reduce patient-borne costs) in patients deemed clinically eligible in a range of conditions, including adult and teenage diabetes [16-18], chronic kidney disease [19], chronic obstructive pulmonary disease [20,21], mental health conditions [22,23], chronic pain [24-26], support after premature birth [27], support of patients in care homes [28], postoperative follow-up for orthopedics [29-32], plastic surgery [33], and prostate cancer [34].

However, all these trials were small; publication bias cannot be excluded, uptake rates were not always reported, and the cost of establishing and maintaining the remote service was rarely measured. Although no specific safety issues or critical events were reported in any of the above studies, exclusion or withdrawal rates in some studies were high [18,23,25].

Notwithstanding the positive findings of randomized trials, audits of actual practice suggest that video outpatient consultations, in common with other forms of telehealth, account for only a tiny fraction of encounters in any specialty [35,36]. Nonadoption, abandonment, and failure of scale-up, spread, and sustainability (the NASSS framework) are the norm when technology-supported service models are introduced in real-world settings for multiple and complex reasons [37-39]. In the words of one critic, the benefits of Skype- and FaceTime-supported outpatient services demonstrated in proof-of-concept studies and experimental trials should be weighed against contextual realities, including “the vagaries of technology, negative views among clinicians, poor uptake by providers, and legal, ethical and administrative barriers” [40].

Some authors have queried whether video consultations might be less reliable [41-43], less safe [44], and less cost-effective than traditional encounters [40]. The online environment is known to produce subtle alterations in the dynamics of human interaction, with a potential risk that clinical clues will be missed or the clinician-patient dynamic altered adversely [45]. The introduction of video outpatient consultations also brings operational and cultural challenges, including the need to develop new ways of organizing clinical and administrative work and train and support both staff and patients in technology use [37,46].

In short, there is a growing mismatch between the positive evidence base emerging from experimental trials and the variable (mostly negative) experiences of teams who try to introduce video outpatient services in the real world [35,36]. The discussion sections of randomized trial reports often suggest that further research is needed into implementation challenges—especially national policy and economic context, the practicalities and costs of organizational change, and the fine-grained detail of how video consultations unfold (in particular, how “quality” and “safety” are achieved and assured) [17,25]. No previous studies have addressed all these issues and their interdependencies rigorously and prospectively. Yet, such research is critical to enrich our understanding of video consulting and to inform and support the development and scaling up of such services. For this reason, we undertook an in-depth, real-world case study of an attempt to introduce video outpatient consultations across several clinical services (in three different hospitals) in a London-based acute trust.

### Aim, Objectives, and Research Questions

Our aims were (1) to define good practice and inform its implementation in relation to video outpatient consultations via Skype and similar media and (2) to generate transferable new knowledge about challenges to scaling up and routinizing this service model in health care organizations.

Specifically, our objectives were as follows:

1. At macro level, to build relationships with key national stakeholders, identify from their perspective how to overcome policy and legal barriers, and create a receptive context for our findings.
2. At meso level, to illuminate and explore the sociotechnical system that supports the video consultation at organizational level and identify how organizations can best support the introduction and sustainability of this service model where appropriate.
3. At micro level, to study the clinician-patient interaction in a maximum variety sample of video consultations and a comparator sample of face-to-face consultations, exploring both effective and less effective communication.

Our research questions were as follows:

1. Macro level: what is the national-level context for the introduction of video outpatient consultations in the United Kingdom, and what measures might incentivize and make such consultations easier to achieve?
2. Meso level: how can a video consultation service best be established, routinized, and sustained?
3. Micro level: what defines “quality” in a video consultation, and what are the barriers to achieving this?

## Methods

### Outline

We have published a detailed study protocol previously [14]; this section provides a summary and refinement of our sampling and analytic approach.

### Study Design

The multilevel, mixed-method study of video outpatient consultations in three hospital departments (diabetes, antenatal diabetes, and cancer surgery: micro level), embedded in an organizational case study of the introduction and rollout of this new service model (meso level), taking account of the evolving national context (macro level), was used.

### Setting

The research took place from 2015 to 2017 in Barts Health, a large, multisite acute trust in a socioeconomically deprived and multiethnic borough in inner-city London, United Kingdom. The organization was under pressure to deliver services more cost-effectively while responding to rising need and demand; outpatient clinics were crowded, and travel to and between its multiple sites was difficult, time-consuming, and (for patients on low incomes) costly.

Clinicians in the diabetes service had been working for several years to establish a remote video option for outpatient consultations to improve accessibility and address high “did not attend” rates. In an early pilot study, we found high acceptance rates by staff and patients for the video option, a significant reduction in “did not attend” rates and small efficiency savings [47,48]. We subsequently demonstrated greater engagement, improved self-management, and better glycemic control in patients with challenging social circumstances and a history of defaulting from appointments who were offered the option of video consultations (see Multimedia Appendix 1 for our 2014 Diabetes Review, Engagement and Management via Skype [DREAMS] report).

This study occurred at a time when the trust was seeking to learn from the diabetes pilot and make video outpatient consultations part of business as usual whenever clinically appropriate. We worked mainly with three services on separate sites in east London: diabetes (adult and young adult), which had been piloting the virtual consultation model; antenatal diabetes, which sought to use such consultations to support close (sometimes daily) monitoring of diabetes in pregnancy; and hepatobiliary and pancreatic cancer, a tertiary care service to which patients were sometimes referred from beyond London, requiring a round trip of several hours. By the end of the study period, other specialties at Barts Health (including neurology, rheumatology, hematology, and endocrinology) had begun to introduce video outpatient consultations.

### Project Management and Governance

The study was delivered by a core working group (TG, SV, JW, JM, and SS), supported by a 6-monthly independent steering group and a patient advisory group (see below). The steering group had a lay chair and cross-sector stakeholder representation, including patients, National Health Service (NHS) stakeholders, and national-level decision makers (details in Multimedia Appendix 2). The study received ethics approval from City Road and Hampstead NHS Research Ethics Committee on December 9, 2014 (reference 14/LO/1883) and subsequent amendments.

### Participants and Data Sources

We collected data over a 28-month period from 36 national-level stakeholders; 24 staff at Barts Health (9 clinicians, 5 managers, 3 technical support staff, 7 administrative support staff); and a total of 50 patients. Data sources are summarized in Table 1 and described in more detail below.

### Sampling and Data Collection: Macro Level

We began with individuals charged with delivering information technology (IT) strategy in NHS England, as well as those leading on patient participation. Alongside review of policy documents (from 2000), we used snowball sampling (asking each interviewee to nominate a colleague) to build up a picture of the national context. We invited a maximum variety sample of 39 stakeholders from across government (eg, NHS England, Care Quality Commission, and NHS Improvement), professional organizations (eg, Royal College of Physicians and Medical Protection Society), patient groups (eg, National Voices), industry (eg, Microsoft), and charitable and third sector organizations (eg, Health Foundation), of whom 36 agreed to talk informally with the study team (3 were uncontactable). We then undertook audiotaped, semistructured interviews with a purposive sample of 12 of these stakeholders, ensuring variation in the different institutions, groups, and perspectives represented. Stakeholder details and interview guides are available in Multimedia Appendix 2.

**Table 1:** Overview of multilevel data collection and analysis in Virtual Online Consultations: Advantages and Limitations (VOCAL) study.

Data source	Type and nature of data	First-order interpretation	Higher order categories
Macro-level study of the wider context for introducing video consulting	Accounts of national-level stakeholders (36 informal and 12 formal semistructured interviews); 50 national-level documents from 2000 onwards (including policies, guidance, and national-level announcements)	Historical and policy drivers for the move to video consultations; system-level blocks	External social structures such as political, regulatory and economic context; background and context to multilevel analysis
Meso-level study of organizational change	Accounts of 24 staff involved in delivering video consultations; approximately 300 hours of observations across 3 clinics; 16 documents (eg, operating procedures and meeting minutes) and researcher field notes about people and technologies delivering video consultations; diagrams and accounts of how people, technologies, and clinical work relate and interact	Key interactions and interdependencies; key organizational routines and how these are changing over time	External social structures (such as professional standards and definitions of excellence, symbolic meaning of illness); internal social structures (what actors “know” and how they interpret the strategic terrain, such as “scripts” held by patients and staff about how they should behave and how they change over time); assumptions built into the technology about, for example, capability of users, how people interact, privacy and consent, the nature of clinical work and routines and how all these interact
Micro-level study of virtual consultations	Video-recording and screen capture (at patient end and clinician end) of 30 virtual consultations (18 diabetes, 12 cancer); field notes from before or after the consultation at patient and clinician end	What is said and done in (video and face-to-face) consultations; unfolding interaction and strategies for communication; how technology shapes and constrains (video and face-to-face) consultations; how participants felt	External social structures (such as professional standards and definitions of excellence, symbolic meaning of illness); internal social structures (what actors “know” and how they interpret the strategic terrain, such as “scripts” held by patients and staff about how they should behave and how they change over time); assumptions built into the technology about, for example, capability of users, how people interact, privacy and consent, the nature of clinical work and routines and how all these interact
Micro-level study of matched face to face consultations	Video-recording of 17 face-to-face consultations (12 diabetes, 5 cancer); field notes from before or after the consultation	What is said and done in (video and face-to-face) consultations; unfolding interaction and strategies for communication; how technology shapes and constrains (video and face-to-face) consultations; how participants felt	External social structures (such as professional standards and definitions of excellence, symbolic meaning of illness); internal social structures (what actors “know” and how they interpret the strategic terrain, such as “scripts” held by patients and staff about how they should behave and how they change over time); assumptions built into the technology about, for example, capability of users, how people interact, privacy and consent, the nature of clinical work and routines and how all these interact
Descriptive and demographic data in the video consultation service	Number of patients offered video consultation option and proportion who accept and persist with it; start and finish time; DNA rate for video and face-to-face options; unscheduled encounters (eg, urgent care) for index condition	Acceptability/popularity of the service; demographic data (eg, uptake by age or ethnicity); failed encounter rate; risk of missing serious problems; consultation length	Background and context to multilevel analysis

### Sampling and Data Collection: Meso Level

The goal of sampling was to map the people, interactions, and organizational routines that support the virtual consultation with a view to building a rich “ecological” picture of the sociotechnical microsystem [49] (and its wider embedding in the organization) needed to make this service model work as business as usual. We began from participating clinics, mapped the individuals and technologies involved there, and then moved outwards to include, for example, finance and clinical informatics departments.

In total, we conducted over 300 hours of observation of consultations and the clinical and administrative work supporting them, combined with semistructured or naturalistic interviews (asking people “on the job” what they are doing and why they are doing it, as people often find it easier to talk about the detail of their job while they are actually doing it [50]) with 24 staff. We also collected 16 local documents (business plans, informal guides, emails, and minutes of meetings) and descriptive statistics from each clinic.

### Sampling and Data Collection: Micro Level

We used audio, video, and screen capture to produce rich multimodal data on a total of 30 virtual consultations and made audio recordings of 17 face-to-face recordings matched for clinical condition (in which the clinician stated the encounter could have happened virtually). Details of these consultations are shown in Table 1. We sought maximum variation in age; ethnicity; and clinical, social, and personal circumstances. It was a precondition of ethical approval that clinicians were able to exercise judgment about which patients to invite to join the study.

Specific exclusion criteria were as follows: no 3G access at home, lack of familiarity (by patient or carer) with the relevant technology, clinical inappropriateness (eg, need for direct physical examination), inability to give informed consent, and comorbidity preventing participation (eg, severe visual impairment). Clinic populations included a high proportion of limited English speakers. In the young adult diabetes clinic, bilingual health advocates were available and trained in the use of remote consulting, so limited English was not an exclusion criterion there. In the antenatal diabetes and cancer clinics, a remote interpreting service was not available, but patients comfortable with a family member interpreter were included.

Our micro-level dataset consisted of video recordings of consultations incorporating two video streams: one from the clinic and one from the remote site (typically the patient’s home). We recorded consultations using a small digital camcorder with wide-angle lens and remote control (Sony Handycam DCR-SR72), mounted on a mini tripod. We used a commercially available screen capture software tool (ACA Systems) run directly from an encrypted Universal Serial Bus (USB) stick to record screen images showing on each party’s computer screen as a video file. A researcher started and stopped the recordings but left the room during the consultation. When the patient used a mobile, tablet, or Mac computer (which could not run the encrypted USB device), the researcher positioned a second digital camera to capture the screen. In 12 cases, the consultation was recorded at the clinic end but not at the patient end for logistical or patient preference reasons.

Each end of the consultation resulted in 2 digital files, one screen capture and one video. We synchronized these into one file using video editing software (Sony Movie Studio)—allowing us to play the video of the computer screen exactly in parallel with a video of the patient looking at the screen and to view the patient and clinician “ends” in parallel. Face-to-face consultations were audiorecorded using a digital dictaphone. Further details of the informed consent process are given in Multimedia Appendix 2.

The micro-level dataset also included contemporaneous field notes from patients’ homes (eg, material circumstances) and the clinic (eg, physical surroundings and use of paper or electronic records). We also collected demographic data (age, gender, and ethnicity) on patients using the Skype option for remote consulting and (for comparison) on the clinic population as a whole over a 12-month period at each clinic setting.

### Theoretical Framework

In our original study protocol [14], we drew on a technology-enriched version of Giddens’ structuration theory [14], which proposes a dynamic and reciprocal link between (1) the external social environment, (2) human interpretations and action, and (3) technologies; it considers how the relationship between these evolves over time, each shaping the others. For example, the theory explores how human action reproduces and reinforces social norms; how societal expectations (including professional norms and codes of practice) influence the “scripts” built into technologies; how technologies, through their functionality and affordances, make some actions possible but others impossible; and how laws, regulatory restrictions, policy priorities, and professional codes of conduct mean that even when an action is technically and physically possible, it is not in reality an option [51].

The health care setting is heavily institutionalized (ie, our behavior is influenced primarily by expectations of how we *should* or *must* behave in this setting rather than simply by economic or personal concerns, such as maximizing efficiency or pleasure). In such circumstances, behavior is often ritualized (ie, we know and play out the roles expected of us as doctors, nurses, patients, and so on). A key question driving our data collection and analysis was how would the technological and material aspects of the remote consultation shape, enable, and constrain the playing out of these institutionalized roles and behaviors.

As the study unfolded, we enriched and extended this initial theoretical framework with additional material on, for example, clinical aspects of the illness or condition, the kind of knowledge that is foregrounded (or backgrounded) by the technology, and commercial and regulatory considerations. The resulting theoretical framework (NASSS) has recently been published [37].

### Action Research

As described in detail previously [14], our study was informed by the principles of action research [52], defined as “a mutual learning process within which people work together to discover what the issues are, why they exist, and how they might be addressed” [53]. Data collected by and with the research team were fed back formatively to inform development of the service (for instance, where appropriate, we sought to support plans for rollout of virtual consultations across the hospital). In the early stages of the study, we held two formative learning workshops to feed back our findings. As the study progressed, we were welcomed into the trust’s existing governance structures (including an outpatient strategy group set up to drive the rollout of virtual consultations and the local information governance department to develop standards and guidance for such consultations) so bespoke feedback meetings became unnecessary. We also fed back emerging findings periodically to national-level decision makers (for instance, relating to national payment systems) both via bespoke meetings and also because a national policy maker with responsibility for NHS IT was on the VOCAL steering group and another worked closely with us on a related project.

### Data Analysis: Macro Level

Interviews with national stakeholders were initially analyzed thematically to provide context for the statements, actions, and interpretations made by organizational actors at local level. We also used interpretive policy analysis [54,55] to identify the key “storylines” [56] shaping policy and debate around remote consultations and to surface inconsistencies and ambiguities between local and national perspectives.

### Data Analysis: Meso Level

Our approach to mapping the sociotechnical health care ecosystem [49] provided detailed data about the logistical and technical barriers involved in introducing and running remote consultation services (in diabetes and cancer clinics, as well as the wider hospital such as IT and information governance departments) and workarounds to overcome them. This included data about issues related to technology, clinic management, administrative processes, patient enrollment, and the exercise of clinical judgment. We used diagrams and narrative as synthesizing devices to draw together a visual representation and linked verbal account of these human and technical interactions and interdependencies.

We also drew on the notion of “organizational routines” [57,58] defined as “recognizable, repetitive patterns of interdependent action carried out by multiple actors” [59]. Routines are how organizational life is patterned, and hence, studying them provides important insights into how innovations such as remote consultations may (or may not) be assimilated in health care and how that assimilation changes over time. We identified the work required (at clinic, departmental, and executive levels) to “routinize” aspects of the virtual consultation service; examined the dynamics within and across different routines; and analyzed the convergence between stated (or proxy) routines, clinician and staff understandings about how to enact it (ostensive routine), and the range of ways in which it is then carried out (performative routine). This allowed us to reveal the tension between current business as usual and the new ways of working implied by a video consultation model.

### Data Analysis: Micro Level

Our initial analysis of micro-level data involved repeated viewing of selected virtual consultations and discussion in our interdisciplinary team (including sociology, linguistics, human computer interaction, and medicine), alongside review of interview data with patients and clinicians. This led us to identify a number of questions relating, for instance, to the ways in which the context of the consultation (often involving patients in their home setting and clinician at the clinic) shaped communication; whether the usual format of the medical consultation (opening, history taking, examination, diagnosis, and review) might shift when conducted remotely; how talk about technology might reorient patient-clinician interaction; and how sensitive topics (such as breaking bad news) might play out differently.

On the basis of these early emerging themes, we sought a methodology to add depth and detail to our findings and identified the Roter interaction analysis system (RIAS), a widely used method for coding medical dialogue [60]. Broadly derived from social exchange theories related to interpersonal influence, problem-solving, and reciprocity [61-63], RIAS offers a validated coding system [60], allowing researchers to systematically quantify the occurrence of different types of talk that occur during medical encounters that reflects accepted patient and provider roles and obligations in a “meeting between experts” [60]. It has been used extensively to analyze face-to-face consultations but rarely in remote settings. We identified one paper (a conference proceeding) that explored the theoretical potential of RIAS in technology-mediated consultations [64], 3 small empirical studies in different clinical conditions [65-67], and a validation study of new RIAS codes for technology-related talk [68].

Roter’s original taxonomy distinguishes three main categories of talk: “task-focused” (eg, application of medical expertise to diagnose and manage disease), “socioemotional” (eg, greeting, building rapport, and showing concern), and “process” (eg, inviting the patient to sit down). In this study, we also used a fourth category: “technology talk” (eg, that the picture is fuzzy), initially introduced by other researchers [66,68] and adapted and extended by our own team. Table A1 in Multimedia Appendix 2 shows the high-level clusters and more detailed categories used in RIAS with examples drawn from our data.

Following a 3-day training course delivered by the team that originally developed RIAS, we applied the RIAS coding scheme to our micro-level data, addressing five questions about the differences between virtual and face-to-face consultations for the same clinical condition:

1. Are remote consultations shorter and more “to the point” than face-to-face ones?
2. How do they differ in the different kinds of (nontechnology-related) talk that occurs?
3. What kind of technology-related talk occurs?
4. What kinds of breaches (misunderstandings, “repairs,” and so on) of talk occur in virtual consultations, when do such breaches occur, to what extent do they matter, and how might they be reduced?
5. How do interruptions (in the patient’s home and/or in the clinician’s office) affect the flow of talk in the virtual consultation?

The assumptions for normal distribution of the data were not accepted (Shapiro-Wilk normality test was significant at *P*<.05). Therefore, Mann-Whitney *U* tests (nonparametric) were used to compare interactions for virtual and face-to-face consultations.

### Patient Involvement Statement

The initial impetus for introducing virtual outpatient consultations in the diabetes service was from service users (many from deprived socioeconomic backgrounds and/or minority ethnic groups) who found it difficult to attend face-to-face appointments. As noted above, the VOCAL steering group had a lay chair and patient representation. In addition, we sought ongoing patient feedback on both the research process and the developing video outpatient services at Barts Health via a dedicated patient advisory group, with 12 patients (and one spouse) representing a wide range of ages and ethnic groups and facilitated by an anthropologist with close knowledge of the local community. This group met three times throughout the study, supplemented with ad hoc contact with individual members (eg, to invite input on lay summaries). In one of the meetings, the patient advisory group was shown (with the written consent of participants) two video clip recordings of virtual consultations as the basis for discussion. In addition, some preexisting lay groups (one antenatal group consisting of 9 mothers and one spouse, a peer support group for gestational diabetes, and a young people’s peer support group for diabetes) and a cancer support charity were consulted to extend the range of patient and lay input.

## Results

### Macro-Level Findings

The external context for technological innovation in the UK public sector is currently extremely challenging. There is a strong policy push to develop UK’s digital economy [69-73], digital government [74-77], and digital health [7,8,70,78-85]. We found no national policy specifically relating to the introduction of virtual consultations. As one senior decision maker said, “There are pockets of success, and there are certain vanguards exploring it, there’s bits and bobs. But there’s not actively a digital fund for telehealth.” Rather, the policy of using technologies to support new service models appears to be folded into other programs such as the NHS Five-Year Forward View (2015-20) [2] and the General Practice Forward View (2016-21) [86]. The former includes the NHS “vanguards” to test 50 local innovative service models supported by a dedicated tranche of innovation funding [87]. An independent review in 2016 of IT in the NHS called for “new national funding to help trusts go digital and achieve maximum benefit from digitisation” [7] and led to the appointment of 12 NHS hospital trusts as “digital exemplars” with generous additional funding for introducing new technologies [88].

Notwithstanding these initiatives, constraints imposed by financial austerity—spending plans, for example, indicate a decreasing share of gross domestic product being devoted to the NHS from around 7.3% in 2016 and 2017 to 6.9% by 2022 and 2023 [89]—have meant that there has been a little slack in supporting the piloting, organizational learning, and extensive groundwork that is often needed to routinize new technologies or practices. Low growth in NHS budgets [81] combined with sustained increases in demand are taking their toll on providers [90-92].

National-level stakeholders and documents depicted technological innovation as imminently poised to deliver efficiency savings in the NHS “at scale” and “at pace” [81,93,94], thereby helping solve the growing challenge of rising demand and falling budgets and also producing “a virtuous circle of economic growth for the UK” [8]. The industry sector (whether global companies seeking long-term strategic partnerships or small- and medium-sized enterprises offering “niche” products) is depicted in these documents as the innovator and producer of “transformative technologies” [81], with the implication that the technologies, once produced, will *drive* transformation of the NHS. The potential time lag between adoption of technology and realization of productivity gains (if any) was rarely acknowledged either by interviewees or in policy documents.

Few interviewees mentioned low digital literacy and digital access. In the United Kingdom, 9% of all citizens (and a disproportionate number of the poor, sick, and elderly) have never used the Internet [95]. The UK government has an active digital inclusion strategy [70,71,96], which appears to be predicated on a “deficit” model (ie, it assumes that with support and training, everyone will be able and willing to connect digitally to public services). An alternative perspective, taken by patient and advocacy organizations, is that digital exclusion has complex underlying causes linked to the social determinants of health (eg, poverty, social exclusion, language, and literacy issues) and that “training” alone will not overcome these [97].

There was broad consensus among national-level stakeholders that the current evidence base for technology-supported new service models is weak. However, there were striking sectoral differences in what kind of evidence stakeholders believed was needed. Industry interviewees expressed confidence in the “fail early, fail often” approach of iterating software design to optimize the use of a technology in a particular setting. Interviewees from regulatory bodies and professional organizations appeared keen on qualitative evidence (they wanted to know more about, for instance, *what makes a good quality remote consultation*). In contrast, clinicians and policy makers placed high value on RCT evidence generated elsewhere but assumed to be transferable to the current setting [2].

The introduction of virtual consultations was viewed by industry informants as a particularly difficult and risky aspect of NHS IT development because it required major changes to service models and institutional embedding. Suppliers lamented slow time frames, “decision making by committee,” “so much duplication,” “consultation about everything,” and the “need for everyone to go out and evaluate every single thing.”

The prevailing emphasis by NHS England and local providers on one-off procurement contracts for particular technologies contrasts with the strategic desire of many industry stakeholders to develop mature partnerships in which industry commits to supporting an evolving service via an evolving package of technology and support. Industry informants were therefore, perhaps reluctantly, prioritizing business initiatives that rested on “off-the-shelf” technologies that could be bought, installed, and made to work with minimal ongoing work to embed, routinize, and sustain them. They saw greater potential, for example, in what one industry executive called the “wellness and wearables market”—technologies that were more or less freestanding and could often be promoted direct to consumers.

A key issue repeatedly raised by interviewees but rarely evident in published documents was how reimbursement for virtual consultations would be implemented. As one senior decision maker in NHS England told us, “we have a drug tariff that does prescriptions very well, but we don’t have anything for digital.”

Our analysis highlighted several strands of work being undertaken by national-level bodies (NHS Improvement and NHS England) to review payment and pricing. The development of a national Innovation and Technology Tariff for England has gone some way to addressing this by removing the need for multiple local price negotiations (instead assuring reimbursement when an approved innovation is used) but is currently limited to 6 specific medical devices or apps and does not include virtual consultation technologies [98]. The need for individual provider organizations to negotiate payment for virtual consultations with local clinical commissioning groups (even as a temporary “pass through” solution) is likely to be a significant barrier to national rollout and might also compromise the long-term sustainability of existing virtual consultation services. The proposed shift away from activity-based funding to capitated payments in the NHS could potentially overcome this problem but is likely to be several years in development [2,99,100].

### Meso-Level Findings

The action research element of the study meant that we experienced first-hand the reality of setting up and delivering the virtual consultation service in a busy public-sector hospital trust. Despite the fact that Barts Health had been an early adopter of virtual consultations and a willing partner in the research proposal, implementation proved far more complex and difficult than anticipated. One key barrier to progress was the extreme pressure on human and financial resources. Clinicians, managers, and technical staff in every department were under pressure; key posts were unfilled or frozen, and clinics were very heavily booked.

Early success of video consultations in the diabetes service had been achieved partly through workarounds (eg, installation and support of Skype on a small number of machines as a “favor”). Mainstreaming this same service as business as usual across the trust—a shift that required many processes and activities to be formalized and managed centrally—was initially strongly resisted by the IT department because it threatened to “open the floodgates” in an overstretched department and risked impacting the network bandwidth. Yet, support from the IT department (to set up machines, troubleshoot, provide on-the-job training, and configure electronic booking systems to log “video” appointments) was mission-critical, as was the development and implementation of an information governance protocol to ensure compliance with legal and regulatory standards around privacy and data protection. The latter proved extremely time-consuming even with support from the research team who worked with key local staff to develop standard operating procedures and help navigate these through the trust’s approval mechanisms. The resulting document (see Multimedia Appendix 2) was reviewed and endorsed by the UK Information Governance Alliance (IGA), who used it as the starting point for developing national policy guidelines on the use of Skype and FaceTime across the NHS. We also developed a patient information leaflet with input from our patient advisory group and a guidance document for staff on setting up Skype services, which was used to support rollout across the trust (see Multimedia Appendix 3).

Although some clinicians embraced the new technology with enthusiasm, many others were unwilling to try it (claiming to be “too busy”). Those who did join the study talked positively in interviews about the high quality of most video consultations and believed (correctly as it turned out) that video consultations were generally shorter than the equivalent face-to-face encounter. However, they also commented that they had had to take on a number of new roles and practices. These included triage (judging a patient’s suitability for virtual consultation), finding appropriate physical space for “private” Skype interactions outside clinic hours, troubleshooting IT, patient setup (ensuring the technology worked and supporting patients with its use), and medical documentation (adjusting to the different ways in which electronic and paper documents and other artefacts were used in consultations).

Virtual consultations appeared to work best for long-term conditions in which the clinician and patient had a preexisting relationship with a high degree of mutual trust and “common ground” when interdepartmental coordination over clinical care was not required, when the need for close physical examination could be excluded in advance, when there was a clear relative advantage to virtual consulting (eg, a history of defaulting from face-to-face appointments or clinical or practical barriers to the patient traveling), when both parties were confident and competent with technical issues, and/or when there was a pressing clinical need to have repeated contacts with the patient. In the (relatively rare) circumstances in which key clinical, technical, and practical preconditions were met, video consultations appeared to be safe and popular with both patients and staff (patients were particularly positive about convenience and not having to take time off work).

**Figure 1:**
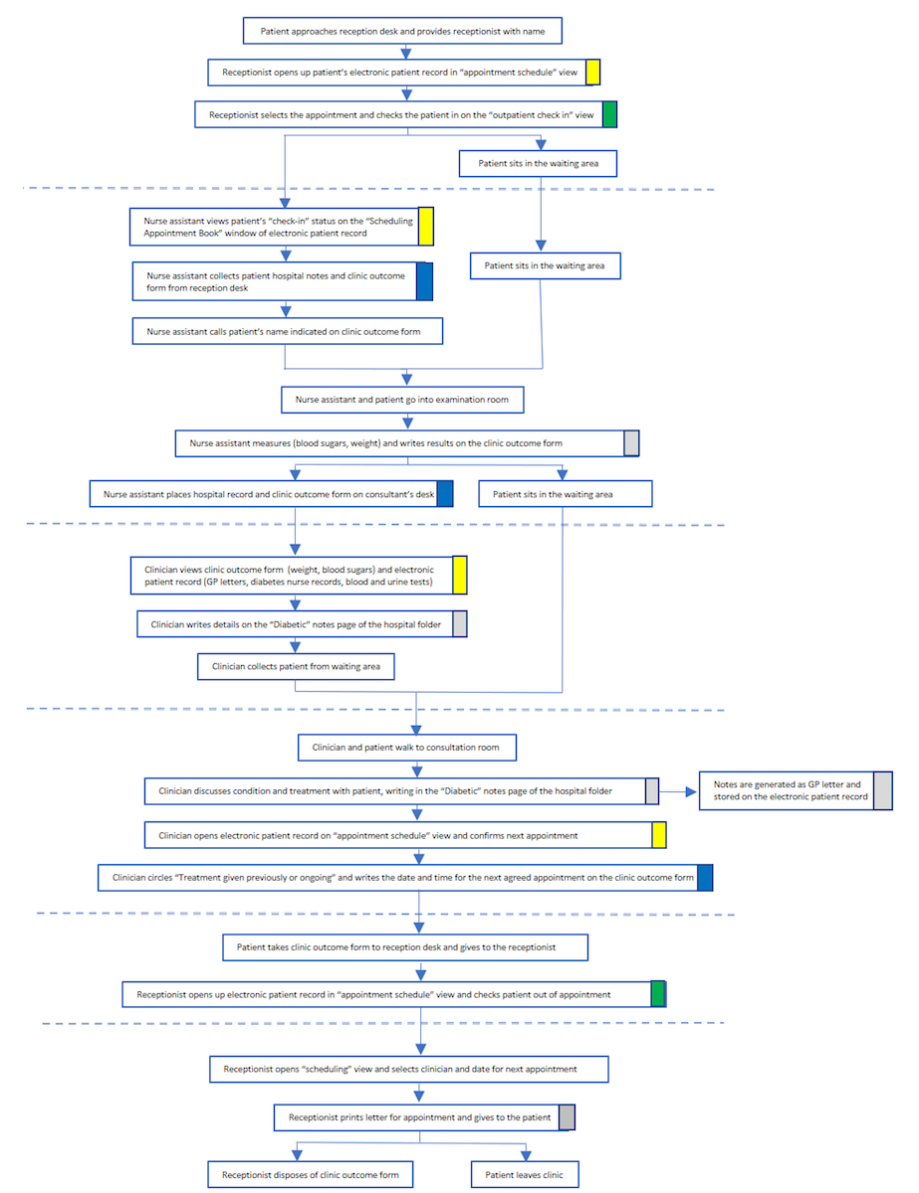
Routine for face-to-face consultation in diabetes adult or young adult clinic.

**Figure 2:**
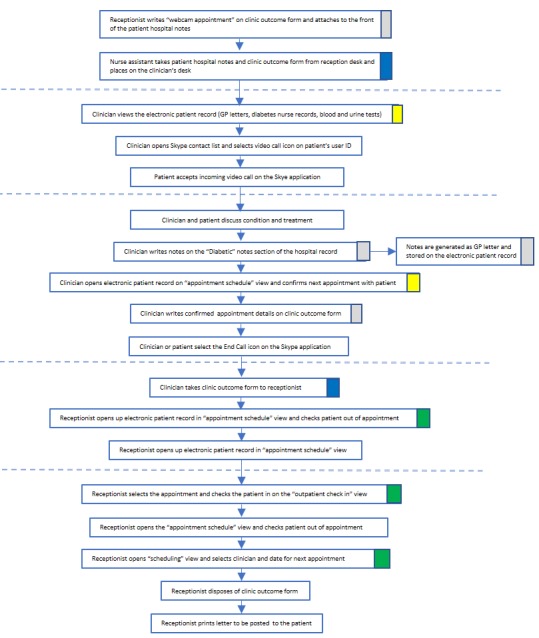
Routine for a virtual consultation in the diabetes adult or young adult clinic.

The many fine-grained differences between the clinic routine for a face-to-face consultation (see example Figure 1) and a video one (Figure 2) illustrate why the process of “embedding” generated both new work for immediate staff and also knock-on effects elsewhere in the organization. Embedding work broadly related to four key processes or subroutines: generating data or information (highlighted gray in Figures 1 and 2), enabling access to data or information (highlighted yellow), facilitating patient access through the clinic (highlighted blue), and tracking the patient through the clinic and care pathway (highlighted green). Crucially, each of these processes was supported by technical and physical artefacts, the movement of artefacts across (virtual and physical) spaces, and the input of multiple clinical and nonclinical actors.

The physical presence of the patient within the clinic setting was fundamental to existing ways of identifying, scheduling, conducting, rebooking, and monitoring patient appointments. For instance, in Figure 1, the physical presence of the patient at reception prompted “check in” and generation of the clinic outcome form: this enabled the nurse assistant to identify the patient, conduct relevant tests, and record the results on the form, which in turn enabled the remainder of the consultation to take place. Embedding virtual consultations in the work of the clinic involved significant reworking of those processes in ways that took account of the “virtual” presence of the patient—as illustrated in Figures 1 and 2 and the additional examples in figures A1 to A4 in Multimedia Appendix 2. The extent to which face-to-face consultation routines needed to be reoriented, and how this reorientation was managed by staff, varied across the three clinic settings, depending on the people, technologies, material artefacts, physical and spatial arrangements, clinical pathways, and assessment procedures already in place.

Another key challenge to the introduction of video outpatient consultations was the reconfiguration of the electronic patient record system to allow the booking of video appointments on the clinic appointment schedules. Each consultant had a “profile” on the electronic record through which appointments were booked by the administration teams. The available appointment types and time slots that could be booked were configured according to their existing clinic schedule. The reconfiguration of the booking schedule to enable the video appointment option was managed by the trust’s information and communication technology (ICT) support department, which developed protocols for the types of appointments that could be integrated into staff profiles and specifications for the payment tariff allocated to these appointments.

The introduction of the video consultation option in any clinical service involved a lengthy process of form filling, enquiries (email or phone), and discussions and agreements among senior service managers.

By the end of the study, the video option had to some extent become business as usual in the diabetes adult and young adult clinic (see Figures 1 and 2) but had evolved in a different way from the original plan. Although only 3.6% of prebooked outpatient appointments for the consultant diabetologist were formally coded on the electronic record as having occurred via Skype, the actual proportion was much higher because not all remote consultations were coded as such. This was partly because a new electronic record system (without a code for remote consultations) was introduced as part of the service part-way through the study and partly because of ad hoc consultations, discussed below.

A significant use of video consultation by Skype was for supplementary clinician-initiated and/or spontaneous patient-initiated encounters (eg, as an ad hoc measure for keeping in close touch with patients who were undergoing a temporary period of instability or heightened need). If ad hoc encounters are added to the denominator, approximately 11% of all outpatient consultations in the diabetes clinic (and 22% of the lead diabetologist’s) were undertaken by video. Clinicians liked the ease with which vulnerable and “hard to reach” patients could send a message via Skype requesting a virtual encounter, allowing prompt clinical input that clinicians believed improved patients’ confidence in self-management, and which in some cases may have averted a serious complication or hospital admission. It was not possible to produce a reliable estimate of the extent to which these ad hoc encounters replaced rather than added to the clinic’s conventional workload or prevented a hospital admission.

In the antenatal diabetes clinic (whose detailed routines are represented diagrammatically in figures A1 and A2 in Multimedia Appendix 2), only one clinician ever used the Skype service (for 2% of her encounters), and it was abandoned after a pilot period. In this (extremely busy) clinic, virtual consultations aligned poorly with a context involving multidisciplinary teams (patients were typically consulting multiple clinicians across departments) with a relatively short-term but high-risk condition (gestational or preexisting diabetes in pregnancy) and in the absence of integrated medical records (paper medical notes being held by the patient and so not physically present at the clinician end).

In the hepatobiliary and pancreatic cancer clinic (a tertiary referral service, figures A3 and A4 in Multimedia Appendix 2), virtual consultations were popular and generally unproblematic for follow-up after cancer surgery (a time when it was neither convenient nor clinically recommended for patients to make a long journey to the clinic). Clinicians reported that the dynamic of consultations was more relaxed (eg, being introduced to family members and pets), and some patients said they preferred to receive bad news (eg, signs of recurrence) in the comfort of their home without the ordeal of a long journey home afterwards. The proportion of all cancer follow-up consultations undertaken via video link rose from 7% to 20% during the course of the study.

There was no significant difference in demographic characteristics (age, gender, or ethnicity) between patients using the Skype option and the overall demographic of the patient population in the antenatal diabetes and cancer surgery settings. For adult and young adult consultant-led appointments in diabetes, there was no significant difference in terms of gender and ethnicity. However, there was a significant difference in age profile, with underrepresentation of patients older than 55 years taking up the Skype option (χ^2^_3_[N=307]=11.7, *P*=.01). Older patients opting for video consultations usually had a technology-savvy younger relative who offered to help.

In all virtual consultation services, there were multiple technical issues to be addressed. These were often easily resolvable, but not all patients (or staff) were sufficiently skilled or confident to undertake the necessary “troubleshooting” to achieve and maintain the video connection or resolve audio quality problems.

The VOCAL study came at a time when senior management at the trust were turning their attention to rollout of virtual consultations beyond a handful of clinics—a factor that was crucial to establishing an outpatient project strategy group (chaired by the chief medical officer) focused on supporting local rollout. That work needed to accommodate competing policy priorities locally and nationally and work with national policy makers, regulators, and industry partners (including the IGA, NHS England, Clinical Commissioning Groups, and Microsoft UK) to find workable ways forward through close dialogue and practical problem-solving.

### Micro-Level Findings

Our micro-level dataset is summarized in Table 2. Key findings are described below.

The opening sequence of a video consultation was very different from that of a face-to-face one. In the former, there was invariably a “technical set-up” phase before clinical talk began. Clinicians sometimes conducted test calls so the patient could familiarize themselves with the Skype technology and/or check that the video and audio worked before the consultation. Clinicians often talked patients through minor technical problems while consulting. Examples of video and face-to-face opening sequences are shown in Textboxes 1 and 2, respectively.

Technical problems included lack of sound, poor sound quality, loss of picture, and patient failing to activate video. All were relatively minor and resolved satisfactorily, sometimes through “workarounds.” For example, poor audio quality in two consultations required patient and clinician to communicate via telephone, muting the sounds while simultaneously running the video display; the problem was later found to be caused by a letter dictation device plugged into the clinician’s computer. Technical issues sometimes led to conversational breaches, typically characterized by concern and/or humor, but the flow of conversation was usually quickly restored (see Textbox 3 for illustration).

Face-to-face consultations were characterized by shared physical space (eg, across the corner of a desk); patients and clinicians typically engaged together with numerous physical artefacts (paper notes, diary, smartphone, insulin pen or pump, scraps of paper, and sticky notes) as consultations unfolded. In video consultations, both parties had to compensate for lack of shared space and artefacts (eg, by holding a page up to the screen or reading aloud from a set of home blood glucose readings).

Apart from technical issues and differences linked to physical layout, video and face-to-face consultations within any specialty were strikingly similar. The content and flow of most video consultations in our dataset appeared to be high quality, though a small fraction appeared awkward and disjointed, with parties frequently misunderstanding or talking over each other and/or needing to seek clarification. Further analysis of these “awkward” consultations is ongoing, but no major safety concerns were identified.

**Table 2:** Overview of consultations in our micro-level dataset.

Clinic	Total recorded	Male or female	Age in years, range (median)	Ethnicity (n)
Diabetes (video)	12	5 male and 7 female	21-50 (23)	White British (5); White other (2); Black Caribbean (1); Asian Bangladeshi (1); Asian Indian (3)
Diabetes (face-to-face)	6	3 male and 3 female	21-58 (26)	White British (2); Black Caribbean (1); Asian Bangladeshi (2); Asian other (1)
Antenatal diabetes (video)	6	6 female	30-37 (34)	White British (1); Asian Bangladeshi (1); Asian other (3); Black Caribbean (1)
Antenatal diabetes (face-to-face)	6	6 female	26-36 (33)	White British (0); Asian Bangladeshi (3); Asian other (1); Asian Indian (1); Black Caribbean (1)
Cancer (video)	12	4 male and 8 female	55-85 (74)	White British (9); White other (1); Asian Indian (1); Black Caribbean (1)
Cancer (face-to-face)	5	3 male and 2 female	45-75 (69)	White British (2); Asian other (1); Black Caribbean (2)

**Example of opening exchange of a virtual consultation for antenatal diabetes. (xx)=length of pause in seconds.**

*Connection established and video display appears on both patient and clinician screen...*

Patient: Ah!

Doctor: Ah hello! (0.53)

Patient: Can’t hear anything. (0.5) Hold on. (1.26) Uh. (2.26) One minute, can’t hear you. One minute, can’t hear you.

Doctor: Are you alright, can you hear me now? (0.04)

Researcher: Can you hear us?

Doctor: I can hear you.

Patient: Is it this one? (0.11) No, no. (1.29) Volume, this one.

Doctor: Hello?

Patient: There it is. Hold on. (0.47) OK, can you hear me?

Doctor: I can hear you, can you hear me.

Patient: Ah, brilliant, yeah.

Doctor: We’re on! Great! How are you?

Patient: I’m fine. Um. (0.27) OK. Um. (0.27) OK.

Doctor: Great. Alright. Now, just looking at what I wrote down at our last meeting, we’d started you on some insulin.

Patient: Yep. (0.04)

Doctor: How’s that been going?

**Example of opening exchange of a face-to-face antenatal diabetes consultation. (xx)=length of pause in seconds.**

*Clinician brings patient from the waiting room to the consultation room, and reads through the patient’s maternity folder...*

Clinician: Right, so we met last time, we’ve met a few times.

Patient: Mhm.

Clinician: So, you’ve had a scan today.

Patient: Yes.

Clinician: How was the scan?

Patient: The scan was good!

Clinician: Was it?

Patient: Yeah.

Clinician: Brilliant, and you’ve seen the baby doctors, what did they say, were they happy?

Patient: Yes, they’re happy, everything is OK, nice growing.

Clinician: Fantastic!

Patient: And they’re preparing for my caesarean. (2.10)

Clinician: So, C section booked for the sixteenth of June!

**Conversational breach related to reduced video quality during cancer surgery follow-up appointment. (()) refers to unintelligible speech.**

Clinician: Sorry—your your uh, the picture has frozen.

Patient: Right (( ))

Clinician: We can hear you very well, but the—

Patient: I can see you moving, (( )) that's fine.

Clinician: Yeah but (( )) your picture has frozen. But a uh—at a very happy expression so we don’t mind.

Patient: Yes [Laugh]

Clinician: [Laugh] Um. (0.39) So we will see you again, or touch touch ba-+base—oh yeah you are moving again, now...

Nurse: That’s better.

Patient: Right.

Clinician: We–we’ll make contact again in November or December, after you’ve had another computed tomography (CT) scan and another set of blood tests.

Video consultations presented new possibilities for interruption. This included disruptions related to the technology (eg, loss of sound and incoming call on the mobile device being used for the consultation), as well as nontechnological interruptions in the domestic environment (eg, family members entering the room). In all cases in our dataset, flow of the consultation resumed readily after such interruptions.

Findings from our RIAS analysis are summarized in Table 3. Consultation length (defined by RIAS as frequency of utterances) was, overall, 13.34% (584/4379) shorter than comparable face-to-face encounters in all three clinical services studied, even taking account of the small amount of initial “technical talk” to establish the connection, constituting 4.43% (168/3795) of all talk during virtual consultations. However, these differences in length were not statistically significant (*U*=121.5, *P*=.43). The extent to which the clinician did more talking (“dominance”) and exerted more control (“directedness”) was similar in both video and face-to-face consultations in each specialty (though it varied across specialties, perhaps reflecting differences in clinical scope and/or clinicians’ consulting styles). The one statistically significant difference in clinician dominance was in the diabetes antenatal setting, in which consultations were slightly more clinician-dominated during remote (median=1.2; interquartile range [IQR]=0.3) than face-to-face (median=1.7, IQR=0.5) consultations (*U*=3.5, *P*=.02). This was probably explained by patients, at the clinician’s request, reading out home blood glucose readings and insulin doses in the video consultations.

A more fine-grained analysis of the different types of talk, which we will present in a separate publication (Wherton et al, in preparation) likewise confirmed only small and mostly nonstatistically significant differences in categories such as “verbal attentiveness,” “making requests,” “giving information,” and “counseling” (see full list of categories in Multimedia Appendix 2); significant differences were again explained by the material circumstances of the consultation.

None of the other differences between video and face-to-face consultations in the above table were statistically significant.

**Table 3:** Median and interquartile ranges (IQR) for clinician and patient talk in virtual and face-to-face consultations, based on Roter interaction analysis system.

Clusters of talk		Consultations, median (IQR)					
		Video			Face-to-face		
		Clinician	Patient	Total	Clinician	Patient	Total
**Diabetes (adult or young adult)**							
	Socioemotional	72 (26.5)	55 (34.0)	120 (51.5)	54 (30.0)	88 (71.8)	117 (71.0)
	Task-focused	82 (38.8)	82 (49.8)	170 (53.5)	122 (24.5)	74 (34.3)	206 (4.5)
	Process oriented	31 (21.5)	3 (4.0)	35 (21.5)	29 (8.3)	7 (9.5)	35 (11.8)
	Technology-related	1 (6.0)	1 (2.8)	2 (8.8)			
	Total number of utterances	181 (42.3)	143 (84.3)	337 (112.5)	204 (38.8)	173 (82.5)	366 (93.8)
	Clinician dominance			1.3 (0.6)			1.3 (0.7)
	Clinician directedness			0.7 (0.5)			0.5 (0.4)
**Antenatal diabetes**							
	Socioemotional	35 (44.5)	38 (39.0)	74 (80.8)	43 (24.8)	36 (26.0)	83 (38.0)
	Task-focused	37 (27.5)	29 (19.0)	66 (48.5)	42 (22.3)	23 (24.2)	73 (30.2)
	Process oriented	6 (8.0)	2 (2.5)	8 (10.3)	11 (12.8)	1 (2.8)	14 (13.3)
	Technology-related	5 (6.3)	3 (3.5)	7 (9.0)			
	Total number of utterances	89 (66.0)	77 (59.5)	167 (125.5)	103 (38.8)	69 (51.3)	168 (76.3)
	Clinician dominance			1.2 (0.3)			1.6 (0.5)^a^
	Clinician directedness			0.8 (1.3)			0.8 (0.9)
**Hepatobiliary cancer surgery**							
	Socioemotional	23 (46.5)	35 (34.5)	77 (35.0)	31 (39.0)	49 (38.0)	71 (72.5)
	Task-focused	42 (40.5)	33 (26.5)	73 (39.0)	70 (38.5)	35 (44.5)	114 (63.0)
	Process oriented	9 (14.5)	5 (6.5)	15 (20.5)	19 (16.5)	4 (5.5)	23 (21.0)
	Technology-related	8 (8.5)	4 (13.5)	12 (22.0)			
	Total number of utterances	108 (148.5)	84 (21.0)	192 (69.5)	137 (62.5)	72 (57.5)	217 (142.5)
	Clinician dominance^b^			1.3 (1.8)			1.4 (0.5)
	Clinician directedness^c^			1.0 (1.6)			0.9 (2.5)

^a^Statistically significant difference between video and face-to-face at *P*<.01 level (Mann-Whitney *U* test).

^b^Clinician dominance=ratio of clinician talk to patient talk (a figure above 1.0 means clinician talks more).

^c^Clinician directedness=ratio of clinician to patient control over consultation (higher number ≥ clinician has more control).

The RIAS analysis did not include the “ad hoc” consultations that occurred in the diabetes clinic (in which, eg, patients sought an immediate, and often very quick, Skype encounter with a clinician known to them to sort out a problem with insulin dosage).

## Discussion

### Statement of Principal Findings 

This study has confirmed findings from randomized trials that when clinical, technical, and practical preconditions are met, video consultations are safe, effective, and popular with participating patients and staff. In most cases, video consultations consisted of similar types of talk, in similar proportions, to comparable face-to-face consultations, and differences between different clinical specialties were more striking than those because of the technology. By the end of this study, between 2% and 22% of all consultations were being undertaken via video link by participating clinicians. In the remainder, the video option was considered impractical, technically unachievable, or clinically inadvisable for the patient. Technical challenges were typically minor but potentially prohibitive.

Although these findings confirm that video consultations may have an important place in transforming care models, some staff members chose not to participate, and patients for whom video consultations were deemed appropriate represented a fraction of the overall clinic workload in all specialties studied.

Notwithstanding policy interest in digital solutions, the reality of establishing video outpatient services in a busy and financially stretched acute trust proved far more complex and time-consuming than anticipated—mainly due to lack of “organizational slack” [101], disruption of traditional clinic routines, and real and perceived information governance challenges.

Although national policy makers viewed video consultations as a driver of change (supporting new, more efficient service models), industry informants viewed this option as low priority because of anticipated (and experienced) challenges of working with the NHS on projects that required complex organizational, policy, and regulatory changes.

These findings can be theorized using our recently published NASSS framework that was developed to explain why, despite significant investment and high expectations, five problems persist: digital technologies are either *not adopted* or soon *abandoned* by professionals and/or their patients and clients or else the technology-supported service succeeds as a small-scale demonstration project but fails to *scale up* locally, *spread* to other comparable settings, or be *sustained* over time. The NASSS framework analyses these problems in terms of seven interacting domains: the condition, the technology, the value proposition, individual adopters (staff and patients), the organization, the external (eg, regulatory and policy) context, and emergence over time [37]. Each domain can be simple (few components, predictable—as in making a sandwich), complicated (multiple components but still largely predictable—as in building a rocket), or complex (dynamic, composed of multiple interacting elements, and unpredictable—as in raising a child).

To the extent that VOCAL was successful in establishing a video consultation service, this was explained by the various NASSS domains: straightforward, predictable, and low-risk clinical conditions; simple and dependable technology that was fit-for-purpose; clear benefits for both the technology supplier and the patient; acceptance of the technology by staff (who considered that the technology supported and extended their professional role) and patients (who were able and willing to develop new skills and ways of engaging); alignment with existing—or emerging—organizational routines; and a strong policy push. To the extent that efforts to introduce video consultations were *unsuccessful*, this can be explained by complexity and unpredictability in the clinical condition; lack of dependability and fitness-for-purpose of the technology; lack of acceptance by staff or patients; limited organizational slack, lack of shared vision, and/or clashes with long-held and difficult-to-change routines; and tricky regulatory or policy issues (eg, national concerns about information governance and lack of a national tariff for virtual consultations).

Our study has also illustrated, through detailed multilevel analysis, the interdependence of the different domains in the NASSS framework. For example, our national-level interviews identified a reluctance among major technology vendors in the United Kingdom (not just Microsoft) to make major investments in partnerships with the NHS. This meant that, at the time of writing, the technology being used was an off-the-shelf product that had not been specially adapted for use in video consultations and that this technology was not high priority for support from the local IT department. This partly explains why significant clinician time (and an extension of the clinician role) was needed to complete such tasks as new appointment booking and management of a virtual waiting room. Our findings suggest that proactive codesign between technology suppliers, participating health care organizations, and national policy makers could potentially produce three things: video software that is more fit for purpose, organizational routines that are better aligned to support video consulting, and better incentives for major suppliers to work in a collaborative and ongoing way with health care providers.

Although our study was not designed to generate a simple or universal “checklist” for implementing video consultations, it is worth reproducing here the five “key recommendations for practice” aimed at clinicians and managers that were coproduced through action research in this study:

- Introduce the service slowly and incrementally with direct involvement of the team to ensure compatibility between the technology and existing practice
- Allow plenty of time for discussion with staff and patients about how it affects the service
- Work in collaboration with your ICT department and technical support teams to establish roles and processes to assist use of the technology
- Use with an understanding of the patients’ lives and how the technology relates to the management of their health condition
- Support flexible use, allowing scope to fit the service around the needs of the patient

### Strengths and Weaknesses of the Study 

To our knowledge, this is the first research study in any clinical field to have taken an in-depth, mixed-methods, and multilevel approach to the study of video outpatient consultations. We succeeded in our goal of collecting rich qualitative data that exposed the “messy reality” of establishing a virtual consultation service and illuminated the pros and cons of using this medium for clinical interaction in different settings. Using action research, we were able to inform and facilitate the work of embedding the new service model and gain detailed insight into organizational complexities and how these changed over time. Working with front-line clinical and technical services, we have developed significant expertise, standard operating procedures, information governance and technical guidance documents, and protocols for setting up and running video outpatient clinics. National policy makers have been engaged from the outset, and the study has attracted interest from other hospitals. A rollout phase continues within the trust, and further work is also ongoing to extend the model to other NHS organizations across the United Kingdom.

The main limitation of the study is its focus on a single (albeit large and multisite) health care organization. Barts Health was even more financially stretched than most acute trusts in the United Kingdom; hospitals under less tight financial and staffing pressures may have found the implementation work easier. In addition, the sample size for the detailed analysis of virtual consultations was relatively small.

### Comparison With Other Studies

Almost all previous research on video consultations in health care has either addressed the technical detail of the remote connection or undertaken an RCT of virtual vs face-to-face consultations [13-18,20-34]. Such studies lend support to the conclusion that in selected patients, video consultations are noninferior to face-to-face ones—but (often by their own admission) they leave unanswered the question of how to establish the service as a real-world option and/or move from a small-scale research or demonstration project to sustainable business as usual. Our finding that, in contrast with concerns raised by previous authors, the technical quality of Skype interactions via available broadband in London was almost always adequate affirms a recent study by others of 4G mobile technology [43].

Some critics will view it as a limitation that we did not emulate the experimental methodology of previous studies. This was deliberate. Our findings have confirmed that virtual consultations cannot be treated like a drug or even as a complex behavioral intervention to be tested “on” patients. Rather, they are the result of a hugely complex sociotechnical system in which “successful” virtual consulting is contingent on multiple factors at multiple levels. If we appear to have produced ambiguous findings, this is perhaps because ambiguity and tension are *inherent* to complex sociotechnical systems. To questions such as “do virtual consultations work?,” “are virtual consultations safe?,” and “are virtual consultations cost effective?,” we suggest the answer will always be “it depends.”

### Meaning of the Study

In the context of a strong policy push to develop digital alternatives to the traditional consultation, delivering video outpatient services at scale is likely to be far from straightforward, as rollout in any locality will be influenced by (among other things) prevailing organizational culture, financial and human resources and priorities for allocating these, existing organizational and technical infrastructure, the nature and causes of professional resistance, information governance challenges, and the logistics of payment. Video consultations, although safe and effective for selected patients, fundamentally change the nature of outpatient care and require clinician buy-in (which may or may not be forthcoming). Industry, although not opposed to the idea of developing software to support video consultations, appears to view organizationally embedded technical solutions as relatively low priority.

The finding that efforts to implement a video consultation service met with multiple challenges in relation to workability and integration aligns with numerous previous studies of other forms of remote care (see in particular Finch and May’s work on telehealth, which formed the empirical basis for May’s normalization process theory [38,39]). Indeed, these difficulties may even have worsened in recent years as clinical work has become more protocolized and financial pressures more severe.

### Unanswered Questions and Future Research

This study has, in some way, revealed and explored the challenges to establishing video outpatient consultations as a real-world service. Overcoming those challenges will not be easy, but further in-depth case studies in both comparable and contrasting settings are likely to enrich our understanding. As more health care organizations make the strategic decision to introduce video consultation services, research could explore the collaboration and mutual learning that occurs between them and test approaches to supporting that interorganizational interaction.

Our macro-level interviews identified a consistent finding from industry informants that the NHS is currently a uniquely difficult setting in which to attempt to introduce technologies that imply major changes in service models. Industry’s preferred model—of long-term partnerships (for technologies plus service support to embed them) rather than one-off procurement contracts—should be introduced in test sites and carefully researched using longitudinal ethnography. The research agenda here is methodological as well empirical; it is founded (we believe) on the notion that technologies and services are continually evolving and mutually shaping; they cannot be fixed in time nor (therefore) be adequately tested using traditional randomized trial designs.

One of the most interesting findings of this study was that the technology provided opportunities for clinicians and patients to use the technology adaptively and differently, allowing new modes of consulting to evolve (eg, patient-initiated contacts direct to the clinician via Skype messaging, which appeared to help supported self-adjustment of insulin dosage in diabetes). Further qualitative research could pursue the consequences of such adaptive usage.

### Conclusions

This study has applied a sociological lens (specifically, an empirically oriented adaptation of Giddens’ structuration theory), as well as the recently-published NASSS framework to a real-world empirical study of video outpatient consultations across three contrasting clinical specialties.

We found that these consultations appear safe, effective, and convenient for patients in situations where participating clinicians judge them clinically appropriate; however, such patients are a fraction of the overall clinic workload. As with other technological innovations, some clinicians will adopt video consultations readily, whereas others will need incentives and support. There are complex challenges to embedding video consultation services within routine practice in health care organizations that are hesitant to change, especially at a time of austerity.

## Appendix

Multimedia Appendix 1 Final report of Health Foundation DREAMS study (2014): Diabetes Review, Engagement and Management via Skype.

Multimedia Appendix 2 Supplementary material.

Multimedia Appendix 3 Skype guidance for clinicians and managers.

## Abbreviations

ICT: information and communication technology
IGA: Information Governance Alliance
IT: information technology
IQR: interquartile range
NASSS: nonadoption, abandonment, and failure of scale-up, spread, and sustainability
NHS: National Health Service
RCT: randomized controlled trial
RIAS: Roter interaction analysis system
USB: Universal Serial Bus
