# Supplementary material for: Real-World Implementation of Video Outpatient Consultations at Macro, Meso, and Micro Levels: Mixed-Method Study
Source: J Med Internet Res. 2018 Apr 17;20(4):e150. doi: 10.2196/jmir.9897 (PMC5930173; doi:10.2196/jmir.9897)
Supplement: Multimedia Appendix 1 [file jmir_v20i4e150_app1.pdf]

Final report

Web-based Consultations in diabetes - a useful tool for supporting patient self-management?

DREAMS – Diabetes Review, Engagement and Management vi Skype

Barts Health NHS

NHS Trust

Part 1: Abstract

The main focus of the DREAMS project was the organisational feasibility of remote ('Skype') consultations and the acceptability of this service model to clinicians and patients in the Diabetes service at Newham.

The project aims were to:

1) *Use online consultations as a tool in establishing contact with those patients typically labelled 'hard to reach', including access, utilisation, impact on experience, interaction and outcomes (including but not exclusively bio-medical)?*
2) *Change the out-patient consultation process for the consultant and specialist nurse involved in this project to enable more flexible online contact as required by patients?*
3) *Use in-depth multi-model methodologies (eg video capture, conversation analysis) and a sociological theoretical framework, explore how a technology-supported remote consultation creates opportunities for new forms of patient empowerment and changing dynamics of interaction around the self-management agenda*
4) *Use social medial platforms to explore the potential for developing online patient support groups with facilitation from health professionals*

The study was designed as a mixed-method organisational case study with three main components: [a] quantitative study of attendance and use of services; [b] clinical case studies of a sample of patients ('index cases'); and [c] qualitative process evaluation of the service.

The study generated large amounts of data, including quantitative data (e.g. attendance at outpatient and A&E, HbA1c, 'do not attend' (DNA) rates, Skype contact) and in-depth qualitative data, comprising clinical case study narratives, interviews with staff and patients, focus group transcripts, and ethnographic field notes. The mixed-methods approach to this research help provided a detailed insight into the potential benefits of using Skype to support patients with diabetes, their experience managing diabetes in everyday life with the support of Skype consultations, and the technical and logistical issues to providing the service. Further analysis is ongoing with a view to producing theorised academic papers.

The research has highlighted the adaptive use of Skype in order to fit consultations around patients' daily lives, which was made possible through the technical functionality of Skype (messaging, 'online status') and their existing relationship with the clinician. This has been reflected in a lower DNA rate for Skype appointments compared to that of face-to-face outpatient appointments.

We also found that patients live very varied lives; some but not all patients get into a good pattern of managing their diabetes and interact well with health professionals. Use of Skype needs to be aligned with the wider social and contextual factors in the patient's lives, as well as their clinical and technical knowledge and capabilities.

A key challenge has been to engage patients in self-management (including the use of Skype or attending clinic appointments). We are now involved in a number of projects aimed at improving self-management and engagement locally, which have evolved as a result of the work

commenced with DREAMS. These include the development of peer-support groups for young people with diabetes and work funded to improve management of patients in the community diagnosed with pre-diabetes.

Skype is now routinely used in the general diabetes and Young Adult Diabetes Clinic, and this will be continued beyond the project. It has been through the coordinated efforts of the staff, and continued collaboration with the Trust's ICT department, that Skype has been able to become embedded within the service.

Part 2. Quality impact: outcomes

This section is intended to explain and review the measures of quality you have used over the last two years and to demonstrate impact from your project. You should address the following points: (around 800 words)

1. **Please describe the primary and secondary data that you used to demonstrate impact on quality, including:**
   a) The source of the data and how easy it was to access (please report against measures being used (as outlined in your measurement plan and analysis page 6, points 1-5) in your proposal)
   b) The validity and reliability of the data
   c) How satisfactory were your baseline numbers in terms of data quality? (If applicable)
   d) What adjustments, if any, have you made to outcome measures from your original application?

The study involved three main components: [a] quantitative study of attendance and use of services; [b] clinical case studies of a sample of patients ('index cases'); and [c] qualitative process evaluation of the service. This section will summarise the data and findings across these components. Further details can be found in the full Research Report (attached).

Quantitative data on attendance and use
For users and non-users of Skype, we collected data on number of appointments (face-to-face and via Skype) and 'do not attend' (DNA) rates. This data was collected using the local diabetes database ('Diamond') and a project database. It included patient details (e.g. age, gender, date of diagnosis), date of the appointments, if they attended or not, if it was a 'patient initiated' 'scheduled', and the duration of the appointment.

Key metrics for unplanned use of services were acute diabetes-related admissions and diabetes-related A&E attendance (even when not admitted). The A&E analysis included both users and non-users of Skype (patients within the same clinic who have not taken up webcam appointments) in the analysis. Historical data for each patient that dates back to their first contact with the Diabetes Department in Newham or Nov 2004 (when the database at Newham was changed), whichever is later, and follow up data to the end of the project or when the patient moved out of the area. This data was obtained from the electronic patient record (EPR). For each A&E

attendance the research nurse reviewed the discharge summaries to identify episodes that were Diabetes related. The relationship between A&E attendance and use of Skype was explored using a Normal-Poisson mixture model.

Patients' HbA1c measures were used to explore change to clinical outcome. HbA1c measures are routinely recorded on the hospital EPR system for each patient. Patients have HbA1c measured at different times for various reasons. For all Skype patients we recorded the nearest HbA1c measure on a 12 monthly basis. The A&E and HbA1c data were obtained from the electronic patient record (EPR), which presented some problems with data retrieval due to the way data is coded and stored within the Trust. This exacerbated by the fact that diabetes services are delivered by multi-disciplinary teams. The research nurses accessed this data via a number of data sources including primary care data through increased work with primary care in the second half of the project.

Clinical case studies

With a view to building a rich picture of how remote consulting either fitted with, or failed to fit with, a person's wider life and their experience of illness, we constructed 16 detailed clinical case studies from three sources: a narrative interview with the patient, extracts from medical case notes (computer and paper) and a semi-structured interview with the doctor and nurse caring for that patient.

The narrative interviews used a conversational style to let the patient set the agenda. They were asked to give a brief account of their background and tell the story of their diabetes and how it was managed. The case note extraction went back to the origin of the hospital record and included biometric data, as well as narrative data. The clinician interview went through each of the 16 patents in turn, in which the clinicians would give their assessment of the patient, their perception of any problems the person had with attendance, and the challenges they perceived in their clinical care.

Process Evaluation

The process evaluation explored the tasks, processes and organisational challenges involved in setting up and running the 'Skype' service, covering clinical, logistical and technical aspects. Informants for this evaluation included patients (n = 15), clinicians (n = 6), administrative staff (n = 3), technical staff (n = 1) and a senior manager (n = 1). The dataset consisted of three patient focus groups, one staff focus group, ethnographic observation in clinic, and a single Skype consultation videotaped from both clinician and patient end. The analysis focused in the identification of barriers and enablers to the implementation of Skype within a clinic and how it was used and embedded within routine practice.

2. **From the outcome measures you have used please share your findings using the data collected (please summarise using run charts, bar charts, tables, or any other format that best shows changes made, for example this could be thematic findings from qualitative measures such as patients questionnaires. You may also want to include short case studies if relevant. (Please include these as appendices).**

Quantitative data on attendance and use

We have observed a lower DNA rate for Skype consultations, compared to face-to-face patient appointments (Figures 1 & 2). A total of 104 patients have used Skype from its inception (2011) to the present. The total number of outpatient appointments with these patients is 1644 with an average DNA rate of 28%. The total number of Skype/webcam appointments is 480 with an average DNA rate of 13% (using scheduled appointments only). A total of 60 patients are currently using Skype. Of these patients, 941 are out-patient appointments with an average DNA rate of 24%. The total number of Skype appointments is 376 with an average DNA rate of 7% (using scheduled appointments only). Further details on service usage are presented in Appendix A.

The Normal-Poisson mixture model did not show a significant difference in A&E attendance under the Skype service compared to the provision on outpatient appointments only. However, a significant difference was observed when controlling for age, in which A&E attendance under the Skype service was 21% than otherwise would be without the Skype option ($p = 0.003$). However, the findings should be treated with extreme caution. As can be seen from the analysis, the results are rather different as different patients and different factors are included. There is a suggestion that the event rate is lower under the web system but there are many possible explanations of this. The sample size is small, as befits a pilot study, and the study is an observational one and not a controlled experiment. Thus the results here should be taken as an illustration of the sort of analysis that might be performed in future studies and not as a valid estimate of the causal effect of offering patients a web-based system. See Appendix B for further details on the statistical analysis.

The HbA1c for current users (N=49) is shown in Appendix C with HbA1c data. The figures show an average HbA1c reduction, in which pre-Skype averaged 70mmol/mol, and end of 2013 averaged 65mmol/mol

Clinical case studies

The in-depth patient case studies illustrated a number of features of this patient group that are expanded on in the patients' own stories. First, the diagnosis of diabetes is life-changing and emotionally traumatic, perhaps especially when it occurs in the teenage years. Second, the experience of diabetes as a long term condition is burdensome, disruptive, time-consuming and stigmatising. Third, patients live very varied lives and cope to different extents and in very different ways with the challenges of diabetes. Fourth, patients experience life events – leaving home, starting college or changing jobs, getting married or divorced, having a child, being bereaved, visiting relatives abroad and supporting family or friends in such life events. The care of the diabetes cannot be separated from these wider aspects of the person's life – which can make clinic attendance difficult for a temporary period. Fifth, some but not all patients establish a positive pattern of proactively managing their condition, attending appointments, engaging with their progress and interacting with health professionals in a way that fits in with their home and work/college life and accommodates the ups and downs of life events. The Skype option helped considerably in some patients but to only a limited extent in others. Our case studies also revealed that rarely, patients with type 1 diabetes exhibit profound lack of engagement with their care and even self-destructive behaviour such as wilful omission of insulin – perhaps as a result of wider

personal, family or social difficulties. In such individuals, the offer of remote consultations was sometimes but not always accepted but when it was, the patient appeared to value the option of unscheduled Skype appointments as it allowed them to make contact on the rare occasions when they felt ready. An example case summary is presented in Appendix D.

Process Evaluation

Skype was used adaptively by patients to fit consultations around their everyday lives and routines, as well as in response to changes in their personal circumstances and support needs. This was made possible through the technical functionality of Skype (messaging, 'online status') and their existing relationship with the clinician. In addition, participants emphasised distinct, but complimentary, differences between online and face-to-face consultations.

The analysis revealed technical and logistical issues that need to be considered to implement and use Skype effectively. These relate to Set-up and maintenance (e.g. NHS connectivity, installing and upgrading software); Managing Skype accounts (e.g. creating clinic accounts, requesting and accepting Skype contacts); Communication via Skype (e.g. 'scheduled' and 'patient initiated' calls, messaging); Procedures around use (e.g. logging on/off, availability status, maintaining privacy, admin), This insights will support development of the guidance materials and SOP document described below.

3. **What impact (from any changes in your data/findings) have you had against your original intended aims? Please also report if there were any unintended consequences of your intervention? If no changes were seen why was this?**

Our findings seem to suggest better outpatient attendance when people have online follow up, probably due to the ease and convenience. The improvement in general outpatient attendance, improvement in blood glucose control and the qualitative date is interesting, and may suggest greater engagement with services, and improved self-management. We are uncertain if this reflects the flexible, user-centred model of care that we have now developed along with a number of other initiatives (developed to address findings from patient interviews and focus groups) aimed at increase patient engagement, rather than the use of Skype-itself. Our findings however support the use of Skype as a tool in providing patient-centred care.

An unintended consequence of using Skype was patients' use of the technology to access clinicians directly and bypass administrative process, mainly for booking or rescheduling of clinical appointments. This raises potential challenges with regard to manageability (e.g. keeping record of upcoming appointments, allocating fixed appointments slots and waiting times) and risk of unequal access (i.e. people with Skype can arrange an relatively quick and immediate appointment face-to-face, whilst those without are restricted to the existing administrative process of fitting into available appointment slots). It may be necessary to manage patients' expectation as to when the clinician will respond or the types of requests that can meet, and consider how this may be addressed through the SOP work.

Part 3: Cost impact.

Please summarise your key cost measures and explain any financial impact the intervention has had over the last 2 years (around 500 words)

- Please describe how you have estimated the cost of existing services / pathways / packages of care. Are there any issues or limitations that need to be taken into account?

- How have you calculated the cost of the Skype intervention? Are there any issues or limitations that need to be taken into account?

Like all long term illness, diabetes care pathways are complex with multiple patient contacts with various members of the multi-disciplinary team, who are employed by different trusts (East London Foundation Trust, Barts Health and Newham CCG in our case).

Diabetes Nursing is provided by ELFT who have an SLA with Barts Health to provide 4 hours/week of specialist nursing for 16-25 year old patients with diabetes. Since the time spent per patient on a Skype-appointment is often less than that when seen face-face, it could be argued this would improve productivity

The tariff for each consultant follow up appointment is £118. The reduction in DNA from a baseline of 33%(general) and 50% (adolescent) clinic to 28% among skype users, with 7% DNA rate for Skype appointments, would suggest significant cost savings as the volume of patients using Skype for follow up increases. Similarly, the average cost of an A & E attendance is £122 with higher costs for patients with diabetes requiring ITU/ HDU stays with diabetic ketoacidosis. The possible impact on A & E attendance is therefore interesting.

An average Skype appointment lasts 9 minutes. This allows an average of an additional 2 -3 patients to be booked per consultant list (in lieu of a face-face slot of 15 minutes). With approximately 53 consultant clinics each year (including the young adult and general diabetes clinic), an addition of 1.5 follow up slots / clinic (on average) will provide approximately 80 additional appointments slots, bringing in income of £9,440 per year to the hospital (£118 tariff consultant follow up x 80), based on current activity levels

As part of the NIHR Leadership Development and Support Programme, T Greenhalgh (academic lead, DREAMS), I Hodgkinson (commissioning lead) and S Vijayaraghavan (clinical lead) met with Ashridge consultancy to discuss ways of determining the true cost of a Skype appointment in diabetes. Discussions suggested a longer term capitated 'Year of Care' tariff was preferable for Skype appointments, and that evaluation of any model of service delivery should include the economic impact on the patient.

We have also had detailed discussion with the NHS Monitor 'pricing team', who are interested in looking at tariffs for online appointments for 2016/17, recognising that current tariffs for non-face-

face contact (based on telephone appointments) are not appropriate for web-based follow up and could act as a disincentive.

Part 4: Learning: effect to date on benefits for patients

Please provide reflections on your learning with regards to the project aims, please address the following: (approx 800 words)

- To what extent did you achieve all that you hoped to at the start of the project?
- What were the enablers that helped you do so?
- Where there were challenges and things that did not work quite as planned
- Is there any advice to others replicating a similar intervention? Anything that you would do differently?

Achievements

Long term uptake and attendance

Our data has shown a lower DNA rate for Skype consultations than face-to-face consultations. Our qualitative analysis revealed that patients often found Skype consultations to be more convenient and less time consuming than face-to-face appointments. It provided them with greater flexibility to fit appointments around their everyday lives. Additionally, patients perceived Skype as a 'safety-net' in case problems arose, and so the Skype option has played a beneficial role, even when they are not actively using it. Our data has shown a reduction in the uptake of new patients for Skype over time. However, there has been a yearly increase in the retention rate 2011 (39%) to 2014 (100%). We suspect this may be partly due to greater understanding of role of web-based care, and patients it is offered to. We wish to explore some of this with PAM.

Greater understanding of service users

The mixed-method approach to this research provided us with detailed insight into how patients live with their diabetes and how Skype can be embedded within the service to support them. Data on patients' use of the system, alongside their personal accounts, draws attention to the ways in which the role and use of Skype is mediated by factors that are specific to the individual, including technical knowledge, support needs, motivation and familiarity with clinician. These issues need to be considered for each patient so that Skype can be used as an effective tool. Patients' use of Skype will change over time, based on their perceived or actual need for support. This means that the service must be flexible and responsive to such changes.

The following two examples highlight this:
One type 2 diabetes patient with bilateral below knee amputations uses prosthesis, and has suffered multiple-problems arising from poorly fitting prosthesis. He was offered Skype-based care as he finds it difficult to physically attend appointments with his wife accompanying him. He is also very afraid of developing hypoglycaemia, particularly at night, as he would need assistance

to get his prosthetic limbs back on (which would involve disturbing his wife) to take some glucose. This therefore leads him to manage his insulin requirements very carefully. He has never used Skype to establish contact with the team and attends annual clinic appointments. In his interview though, he was very grateful for the Skype "safety-net" should he need it.

Another patient with type 1 diabetes using an insulin pump, had a miscarriage during pregnancy a few years ago. During a recent pregnancy, she had 42 brief Skype-based self-initiated nursing appointments as she struggled with insulin requirements and blood glucose control. She delivered a baby girl without any problems, and felt pregnancy was more positive experience this time.

The data has also allowed us to identify and explore different categories of patient groups, in order to better understand how to engage those who are 'hard-to-'reach', including:

- Those who fail to routinely attend any primary or secondary care appointments
- Those who keep in touch with their GP occasionally but fail to attend hospital appointments
- Those who use Skype and telephone contact with the specialist nurse as required by them, but fail to physically attend doctor-led outpatient appointments
- Those who use a combination of non-face-face and face-face contact with both doctor and specialist nurse

We are taking these insights forward to determine how assessment tools and processes, such as the Patient Activation Measure (PAM), could be used to enhance patient engagement in management of their diabetes (see Appendix E for more details).

Patient information:

We produced Information leaflets for patients, providing information about the Outpatient Appointment. We now post them to all patients by mail with their appointment letter. The leaflet design and content was developed with direct input from patients. In addition, specialists at Moorfield's Eye Hospital provided input on the design to ensure that it was clear and readable for people with impaired vision. Patient feedback on the information leaflets was obtained through interviews and a questionnaire. The questionnaire was developed in consultation with patient and user volunteers at the Well London project funded by the Big Lottery (see a copy attached in Appendix F). Overall patient feedback was positive. All patients involved in the feedback sessions (N=7) rated the information as 'easy' or 'very easy' to understand and the content as 'useful' or 'very useful'.

Online patient forum

We have developed a local online forum for diabetes patients to offer advice and peer-support. The forum was developed with the continual and direct involvement of diabetes patients, through focus groups, usability feedback sessions and a closed beta pilot with volunteer patients. It is important to ensure that such forum is useful, useable and appropriate for patients, and so we have made efforts to involve potential users throughout the development process. We are hopeful that this will be accessed by those patients who do not attend specialist or community services,

providing them with diabetes support and an initial route into accessing local diabetes services, if they so desire.

Enablers

We found that the role of service staff and their relationship with the patient makes the technology ‘work’ in practice. Patients emphasised that it is often the ‘person behind the technology’ that made Skype work so well. They talked about the importance of familiarity with the clinician and other service staff members. In addition, it has been through the coordinated efforts of the staff that Skype has been able to become embedded within the service as ‘business as usual’, through the adaption of work routines in order to accommodate the technology.

The involvement of key stakeholders including primary care commissioners and hospital managers was ensured funding of the service and staff time. This was reinforced through regular communication and presentation of findings at regular meetings.

Working with primary care through the second half of the project ensured feedback about patients who did not seek specialist care.

Challenges

Getting patients to engage with self-management (including the use of Skype or attending clinic appointments) and participate in focus groups or surveys remains a challenge. We have started making changes to clinical service and initiated new research, to address this.

Newham University Hospital went through re-organisation and redundancies, due to large financial deficit following merger into Barts Health, soon after the project commenced. This often led to delays and disruptions.

Concerns about data quality on hospital systems due to coding errors led to the use of various data sources including local databases held by staff involved in the project
.

Advice and future direction

Skype has become ‘business as usual’ within the team that pioneered the webcam consultations. Appointment-making and records management for Skype appointments have been seamlessly adopted by the existing staff. We plan to develop SOPs to support sustained use and facilitate uptake across other departments (see Appendix G for further details)

Beyond this small group of pioneers, Skype usage was informally supported by a number of support staff. For example, the IT department allowed the use of Skype. This could have been a major stumbling block – there is an incentive for any IT support team to keep the number of

supported applications as small as possible. However, it is clear that the Skype consultations have not yet become “business as usual” beyond the team of pioneers: there is no service level agreement (SLA) that governs how quickly Skype updates should be installed on users’ machines for example – thus the team are dependent on the goodwill of IT services. Likewise, the process of charging for Skype appointments has yet to be ironed out with commissioners.

The next stage of this project will need to put these consultations on a formal footing within the organisation. This will include setting SLAs for the consultations as well as SLAs for technical support and formalising the way that Skype support is charged for.

Part 5: Plans for sustainability and spread

This section is intended to communicate your plans for sustainability and spread (up to 500 words). You should include:

- How realistic will it be to sustain the benefits of the project beyond December 2014?
- Please outline how you have spread into other services to date and what are your plans for further spread?
- Please detail any external interest/potential contacts that you have already engaged with – what has been the impact of this?
- Please detail any external interest/potential contacts that you have identified that you need to pursue in the future – how will you engage with them?
- Please detail any external interest/potential contacts that you have identified that you need to pursue in the future – how will you engage with them?
- Please list any awards you have won, conferences spoken at etc

Sustaining benefits beyond December

Web-based follow up appointments are now routinely used in the general diabetes and Young Adult Diabetes Clinic, and this will be continued beyond December 2014. The work is well supported by Barts Health and the local CCG, with active support from, service managers and clinical directors. We therefore expect it to continue. We are also involved in a number of projects aimed at improving self-management and engagement locally, which have evolved as a result of the work commenced with DREAMS. These include the development of peer-support groups for young people with diabetes (funded by UCLP) and work funded to improve management of patients in the community diagnosed with pre-diabetes. Use of Skype in patient engagement and management is a key part of both proposals.

We are somewhat concerned that Teresa O’Shea, the diabetes nurse lead for this project, and the largest user of Skype in the follow up of young adults has taken early retirement on 30 November 2014. We are however reassured by the fact, the two part–time nurses replacing her, are both trained in the use of Skype with one of them, Rita Sudra, being the research nurse for

the DREAMS project! We have assurance from the management team at ELFT that these and all other community nurses that web-based care will continue to form part of patient management

Spread to other services

Barts Health

- Skype-based follow up is now being used for the management of patients with thyroid disease by a colleague Prof SV Gelding and her specialist registrar. The project is being evaluated by the BMJ Quality Improvement programme, and was a finalist poster for the UCLP Quality Forum September 2014.

- Skype-based follow up is also being used with the HPB cancer service, a tertiary-referral service, for the post-operative management of patients

ELFT

- All community diabetes nurses have now been trained with access to Skype, with plans for use in routine diabetes management from 2015

We have also been funded by the NIHR for a two-year programme VOCAL (Virtual online consultations: advantages and limitations) to commence February 2015.The project aims to explore online consultations at three levels:

- *Micro, clinician-patient dynamics:* an in-depth qualitative study of the clinician-patient interaction in a maximum variety sample of 30-45 outpatient consultations in two clinical areas (diabetes and cancer)

- *Macro, spread across the organisation and its impact:* Identifying how organisations can best support the introduction and sustainability of this service model in areas where it proves acceptable and effective.

- *Macro, national strategy:* build relationships with key stakeholders nationally and identify from their perspective how to overcome policy and legal barriers to the introduction of virtual consultations

External contacts we have engaged with

- Luke Guiness, Forster Communication, is writing a blog/article for healthcare trade media, to increase awareness of the role of Skype in patient management

Staff from Forster Communication have also interviewed and filmed team members and patients for material, that will be placed on the Health Foundation website as resource material

- A number of external staff have been in contact (list off staff/departments listed in Appendix H). We propose to send them a summary of the final report and SOPS/guidance for the use of Skype in clinical care, when it is developed
- Working with the Co-creating Health Project team at the Whittington Hospital has allowed SV to complete the Advanced Development Programme in 2014
- We have been in communication with Insignia Health, who would be happy to help us with providing licenses for the PAM work

External contacts to engage with in the future

We have been in contact with Bob Gann, Programme Director, Widening Digital Participation Patients & Information, NHS England who has now put us in touch with Roger Donald, the NHS Choices lead for digital assessment, who is keen to explore further the use of VOIP and video technology to assist in patient and clinician consultations.

We are now developing guidelines and a standard operating procedure (SOP) to support other NHS departments and organisations implement and use Skype within clinic services. We will be developing these resources in collaboration with the Barts ICT office to provide technical and logistical guidance (see Appendix G for details on the planned activity for SOP development).

SV has been invited to join a working group set up by the two Clinical leads for Young People and Diabetes, which will explore initiatives to improve user engagement, including the use of online care. The team is meeting 14 January 2015.

Awards and dissemination

Barts Health was visited by the Care Quality Commission in November 2013 and the report published in January 2014 highlighted the role of Skype-based follow up appointments provided by us in the diabetes clinic as an area of good practice by the Trust. This was acknowledged by the Trust Board following this.

Presentations:

Internal:

1. Barts Health Trust Board
2. Trust Transformation Board which is exploring new ways of working which address the dual challenges of improved efficiency and improved patient engagement.
3. Digital Health Symposium

National:

1. The Young Endocrine Diabetes Federation Debate: The remote diabetes consultation: telephone, internet and virtual consultations are a valid substitute for in-person doctor-patient communication.
   For: *Dr Shanti Vijayaraghavan, Newham Diabetes Team,* Against: *Dr Frank Joseph, Countess of Chester Hospital.*
2. DAWN: Diabetes Appointments via Webcam in Newham – "Showcase and Share" session at the Diabetes UK APC 2014. The event was introduced by Dr J Valabhji, National Clinical Lead for Diabetes. This was followed by a lot of external interest from the National diabetes community
3. We were requested to contribute to the NHS Innovations Case Studies document in January 2014
4. Visit from NHS Commissioning Board (Luke O'Shea, Head of Patient Participation & Henry Pares, Patient and Information, NHS England) 2013
5. Invited speaker UCL Partners Quality Forum September 2014 which had Sir Bruce Keogh, National Medical Director, NHS England as keynote speaker.
6. STAR/ Skype Thyroid Assessment and Review; UCLP Quality Forum 2014
7. ABCD (Association of British Clinical Diabetologists); "Young Adult Diabetes Services- What do patients think of them" Invited Speaker October 2014

International:

1. IPIHD Latin America Forum/ Diabetes Steering Group (http://www.ipihd.org/): Improving Patient Engagement in Diabetes, November 2014
2. Invited to create Delegate Experience Day for BMJ/IHI Forum October April 2015

Awards:

1. ISQua Edinburgh 2013: Best Poster awards
2. Quality in Care Diabetes 2013- Patient Engagement category
3. E Health Industry 2013: National Finalists – Best use of IT to support clinical care and management
4. Mary Secole Nursing Award to Desiree Campbell-Richards, Research Nurse (DREAMS) for her work with patient engagement. The work is being written up for her PhD
5. NIHR Research Capability Fund to support salary costs for Joe Wherton, social scientist, to work on DREAMS
6. VOCAL: Virtual Online Consultations- Advantages and Limitations. NIHR HS & DR funding 2014, co-applicant
7. Developing Peer-Support groups for Young People with Diabetes; GUttman Academic Collaboration. UCLPartners 2014, PI
8. Pre-Diabetes: Definition, Screening and Management. UCLPartners, 2014, PI.
9. NIHR CLAHRC Children and Adolescent's Theme, co-applicant 2014

Publications:

- Online Outpatient Consultations: the patient perspective. Poster at Diabetes UK 2014
- Innovo Age January 2014: Case Study
- Contributions to Trust newsletter and trust research bulletin
- Diabetes Appointments via webcam in Newham; invited publication submitted to British Journal of Vascular Disease October 2014 – awaiting outcome
- The work continues to be referenced in the lay press; the most recent article was in the Times newspaper 15 October 2014 ("Your medical data has been loaded – your doctor will skype you now").
- Abstracts have been submitted for poster presentations to the Diabetes UK APC 2015 and the International forum on the Quality and Safety in Healthcare April 2015.

Part 6: Project finance.

The Budgetary Statement (as at 30 November 2014) from our finance department is attached with the report. The budget balance for this date is £62,163. The reports are prepared using extracts from Trust's finance system. This means that transactions that are not registered on the system (invoice paid, money received in the account etc.) as at month end 30/11/2014, will not be shown on the reports. Therefore, we have presented the current budget and expected transactions in Table 1. The expected budget balance once these transactions are complete will be an under spend of: £38,674.

Table 1: Expected balance by 31/12/2014

Description	Cost	Expected Balance
**Budget balance as of 30/11/2014**	-	**£62,163**
Staff time: *Teresa O'Shea*	£2,897	£59,266
Staff time: *Tracey Partlett*	£362	£58,904
Invoice: *Emma Byrne*	£10,000	£48,904
Invoice: *Westfield vouchers for participants*	£200	£48,704
Invoice: *Webcams x2*	£30	£48,674
Invoice: *Statistician consultancy*	£10,000	£38,674
**TOTAL**	**£23,484**	**£38,674**

As previously discussed we plan to use the under spend to take forward some of the work that has emerged from the DREAMS project. The first project will further pilot the Patient Activation Measure (PAM) to study how we can support patient engagement with the service and the role of Skype in supporting these patients (see Appendix E for further details on work plan and cost

details). The second piece of work will focus on developing an SOP for Skype to be used within the Trust and other NHS services (see Appendix G for work plan and cost details).

In addition, we plan to carry out further activity in 2015 to support dissemination and sustainability of project outcomes. This includes a dissemination event in March 2015, filming of volunteer patients and purchase of 50 webcams that can be used by other NHS departments who are interested in using the Skype option. The total cost for these activities is £24,725 and would leave an under spend of £13,949 (Table 2).

Table 2: Expected costs and balance for Jan-Mar 2015.

Details on salaries/service	Cost	Expected balance
PAMS pilot work	£12,975	25,699
SOP development work	£5,000	20,699
Dissemination event	£5,000	15,699
Patient filming	£1,000	14,699
Webcams x50	£750	13,949
**TOTAL**	**24,725**	**£13,949**
