# Supplementary material for: Real-World Implementation of Video Outpatient Consultations at Macro, Meso, and Micro Levels: Mixed-Method Study
Source: J Med Internet Res. 2018 Apr 17;20(4):e150. doi: 10.2196/jmir.9897 (PMC5930173; doi:10.2196/jmir.9897)
Supplement: Multimedia Appendix 2 [file jmir_v20i4e150_app2.pdf]

# Supplementary material

This appendix contains:


1. Membership of the study steering group (page 1)
2. Details of national-level stakeholders interviewed (page 2)
3. Topic guide used for national level stakeholder interviews (page 3)
4. Details of informed consent process (page 4)
5. Patient information sheet (page 5)
6. Patient consent form (page 8)

And

Table A1: RIAS clusters and categories illustrated with examples from our data (page 9)

Figure A1: Routine for a face-to-face consultation in the Antenatal Diabetes Clinic (page 11)

Figure A2: Routine for a virtual consultation in the Antenatal Diabetes Clinic (page 12)

Figure A3: Routine for a face-to-face consultation following hepatobiliary cancer surgery (page 13)

Figure A4: Routine for a virtual consultation following hepatobiliary cancer surgery (page 14)


## 1. Membership of the Study Steering Group

Satya Bhattacharya, Consultant Surgeon at Barts Health NHS Trust

Jenni Bowley (Chair), Citizen / lay representative

Emma Byrne, Researcher in Health Technology at Queen Mary, University of London

Desiree Campbell-Richards, Research nurse at Barts Health NHS Trust

Anna Collard, Social Anthropologist / Independent Consultant

Trish Greenhalgh, Professor of Primary Health Care at University of Oxford

Charles Gutteridge, Chief Clinical Information Officer at Barts Health NHS Trust

Philippa Hanson, Consultant Physician at Barts Health NHS Trust

Isabel Hodkinson, GP Principal and Clinical Lead at Tower Hamlets CCG

Geraint Lewis, Chief Data Officer at NHS England

Joanne Morris, Research Manager at Barts Health NHS Trust.

Seendy Ramoutar, Clinical Nurse Specialist at Barts Health NHS Trust

Sara Shaw, Senior Researcher at University of Oxford

John Taylor, Citizen / lay representative

Shanti Vijayaraghavan, Consultant Physician (with special interest in Diabetes) at Barts Health NHS Trust

Helen Wenseley, Divisional Manager Medicine at Barts Health NHS Trust

Joe Wherton, Senior Researcher at University of Oxford

## 2. Details of national level stakeholders interviewed

| Organisation/group | Exploratory interviews | Semi-structured interviews |
|---|---|---|
| Industry, including small and medium sized enterprises (SMEs) and large companies | 7 | 4 |
| Organisations and regulatory bodies with a concern for quality, safety and governance issues relating to digital health services | 7 | 2 |
| National policymakers, with an active role in developing digital health in the NHS | 7 | 2 |
| NHS executives with an active interest in digital health and/or virtual consultations | 4 | 1 |
| Patients and/or public, either using or representing those using digital technologies | 4 | 2 |
| Professional bodies, including Royal Colleges | 4 | 1 |
| Researchers/knowledge brokers working in the field of digital health and care | 3 | 0* |
| Total | 36 | 12 |

* Guided by our initial analysis of exploratory interviews, we did not invite those whose primary role was researcher/knowledge broker; however, at least two of those sampled in other categories also fulfilled this role.

## 3. Topic guide used in formal interviews with national level stakeholders

1. Can you start by telling me about your background and current role? Especially how it relates to telehealth /remote consultations

2. Who do you / your organisation interact with when you're doing your job relating to [telehealth/remote consultations? Can you give me an example of how that interaction plays out in practice?"

3. What in your view are the drivers for telehealth/remote consultations? What do you think is driving it forward and what do you think is holding it back?
    - Which papers or other documents do you think of as guiding policy in this area? What do you think of these documents?

4. How has policy to promote remote consulting been received by your organisation to-date?
    - How/has it been operationalised within your organisation? Examples?

5. If you were going to change policy for developing and implementing remote consultations, what would you do?
    - What aspects of the current system work really well?
    - Can you give me an example?

6. What do you see as the main challenges nationally to scaling up remote consultations, where clinically appropriate?
    - Commissioning/procurement, reimbursement, technology standards / interoperability, digital participation / access

7. How is your organisation trying to address these? Are you aware of any other activity that's going on to address these?

8. [if not raised spontaneously] What in your view are the information governance challenges to remote consultations? What activity is going on in [your organisation] to address these?
    - Are you aware of any other activity that's going on to address these?

9. Is there something else I should be asking you or other people involved in this project

THANK YOU.

## 4. Details of informed consent process

Care was taken to obtain and maintain informed consent. Information sheets and consent forms (see Appendices 4-9) incorporated guidance issued by the General Medical Council on the video-recording of consultations for research purposes, including an opportunity to withdraw consent after the consultation.* When recording video consultations, the researcher arrive at the patient's chosen venue (usually their home) at least 30 minutes before the booked time slot so as to explain the procedure again, confirm consent and get this in writing, and informally discuss the patient's hopes, fears and expectations for the consultation. We sought similar consent from the health professional at the clinic end of the consultation.

* GMC guidance for doctors: Making and using visual and audio recordings of patients. London: General Medical Council. http://www.gmc-/uk.org/guidance/ethical_guidance/making_audiovisual.asp#Recordings.

## 5. Patient information sheet



Barts Health NHS
NHS Trust

Barts and The London
School of Medicine and Dentistry

# Would you like to take part in research to help design online appointments for people with diabetes?

### Study title: VOCAL - Virtual Online Consultations: Advantages and Limitations

**Invitation and brief summary**
We are a research team from University of Oxford, Barts and the London School of Medicine and Dentistry and Barts Health NHS Trust. We are studying whether Skype can make appointments with your diabetes care team more convenient and more helpful for you. We don't yet know whether the technology is right for this sort of consultation so we want to try it with a few volunteers first and ask them about their experience.

**Purpose of and background to the research**
Our research team, which is led by a GP, is looking at the way in which online communications technology can help people with diabetes. We know that visiting the hospital for appointments can often be time-consuming or inconvenient and we would like to offer a way of "attending" these appointments by Skype. However we need to be sure that these Skype appointments are at least as good as a face-to-face appointment when it comes to the standard of care that you receive.

In order to understand the potential advantages or disadvantages of Skype consultations, we are conducting a detailed study of the patient-clinician interaction through such technology. We are inviting patients to use Skype to communicate with their clinician and tell us their views and experiences of using the technology. With your permission, we will video record one of your Skype consultations to help us understand how Skype can affect communication between patient and clinician. This research will help us understand how Skype can be used to provide better care for people living with diabetes.

You don't have to agree to be part of this research. If you decide not to take part it won't affect your care.

**What would taking part involve?**
Participation in the study will involve using Skype to communicate with your clinician. You and your clinician will decide together when to conduct consultations via Skype. It is completely up to you as to when you use the Skype

option. If you wish to have a face-to-face consultation at any point, instead of using Skype, then you can do so and resume the Skype option at any time during the study.

We want to study the experience of using Skype from the patients' perspective. A researcher will visit you at home during one of your consultations in order to observe how you find using Skype and ask your views about the advantages and limitations of using the technology. You can invite whoever you want to be with you when the researcher visits you.

The researcher would visit you at home to video record your Skype consultation. A video camera will be positioned unobtrusively near your computer screen to capture your interaction with the clinician. The researcher will start and stop the recordings but will leave the room during the consultation. The researcher will not interrupt your consultation until you call them back into the room. When called back into the room, the researcher will check that you are still willing for the video material to be used in the research.

During the visit to your home, the researcher will interview you to find out more about your experiences with using Skype. During the interview, the researcher will ask you about your experience managing diabetes, your views about how Skype can support communication with your clinician, and ways in which the technology or service could be improved. With your permission, the researcher would like to tape record the interview.

The video and audio recordings will be anonymised before anyone else sees it – your name will be "bleeped" and your face and other identifying features will be blurred. The visit will last about one hour in total.

With your consent, the researcher may also want to view selected parts of your medical record or nursing record to see how your diabetes is controlled.

**What are the benefits to taking part?**
You will not benefit directly from the findings of the study, though we hope that the study will help us understand how Skype can be used to support and enhance care of people with diabetes.

**What are the potential disadvantages and risks of taking part?**
We understand that the time commitment and the process of having a researcher in your home can be potentially burdensome. The visit to your home will be approximately one-hour. The date and time for this visit will be completely up to you. If you decide to cancel or reschedule the visit at any point then that is absolutely fine. Participation in this study will not affect your standard care. You are still able to have face-to-face consultations with your clinician at any point during the study.

**Why have I been invited?**
You have been invited either because you have expressed an interest in using new technologies to help manage your diabetes or because your doctor or nurse, carer has identified you as someone who might wish to do so.

**Do I have to take part?**

No. Participation is entirely voluntary, and you can change your mind about taking part even after the study has begun.

**What do I do next?**
If you would like to be involved in the study, please read the consent form and sign it.

**Is it confidential?**
All information you give is completely confidential. We won't tell anyone that you are speaking to us, though you are welcome to tell them yourself. All information will be held securely in strictest confidence. The data will be stored with its own unique identifying code and not with the participant's name or corresponding linkage details, which will be stored separately. Only those directly involved in the research will have access to the data.

The research team may wish to use sections of the video recording for presentation purposes (e.g. training events, conferences, online publications). If you do not wish for the recordings to be used for presentation purposes then that is absolutely fine. You can indicate whether or not you would like us to use the video recording for presentations and/or publications on the participant consent form. If you do give permission for your video recording to be used in this way, we will still remove your name and all other identifiable information.

**Who is funding the study?**
The study is funded by The National Institute for Health Research (NIHR) under the Health Services and Delivery Research Programme, which aims to improve the quality, accessibility and organisation of health services in the UK.

**Further information**
The study team is led by Professor Trisha Greenhalgh, a medical doctor at University of Oxford who is also custodian of the study information. The other members of the team are Shanti Vijayaraghava (Diabetes Consultant, Barts Health), Satya Bhattachrya (Consultant General and Surgeon, Barts Health), Joanne Morris (Research Manager, Barts Health), Sara Shaw (Researcher, University of Oxford), Emma Byrne (Researcher, Queen Mary University of London) and Joe Wherton (Researcher, University of Oxford).

Thank you for your help!

The VOCAL research team

## 6. Patient consent form

VERSION 4_17 August 2016

Barts Health NHS Trust | Barts and The London School of Medicine and Dentistry

# VOCAL RESEARCH STUDY CONSENT FORM

Please initial all the boxes and give this form back to the person who gave it to you. If you don't feel able to initial all the boxes, or if you change your mind at any point, you can choose not to take part in the study.  Whether you take part or not, this will not affect your care in any way.

| | Initial |
|---|---|
| I have read the information sheet Version 5 dated 17 August 2016 and asked any questions I want. | |
| I understand that the VOCAL study is being conducted by researchers from Barts and the London School of Medicine and Dentistry, University of Oxford and Barts Health NHS Trust. | |
| I understand that the research will include the following:<br>• The researcher will video one of my consultations in my home.<br>• The researcher will interview me and ask how I feel about the consultations via Skype | |
| I give permission for my consultation to be video recorded | |
| I am happy for sections of my consultation video to be presented (e.g. conference presentations) so long as my name isn't mentioned and my face is not visible | |
| I am happy for sections of my consultation video to be presented (e.g. conference presentations) with my face made visible, so long as my name isn't mentioned. | |
| I am happy for sections of my consultation video to be presented on online publications so long as my name isn't mentioned and my face is not visible | |
| I am happy for sections of my consultation video to be presented on online publications with my face made visible, so long as my name isn't mentioned | |
| I give permission for my interview to be audio recorded | |
| I am happy to be quoted (for example, when the research is published) so long as all identifying information will be removed. | |
| I understand that if I change my mind about this research, I can say so at any time and the data will be erased and not used further and the research team will no longer contact me. | |
| I understand that the researcher may want to view selected parts of my medical or nursing record. | |
| I agree to participate in the study. | |
| I give permission for direct quotations to be used in reports and publications as long as all identifying information will be removed. | |
| I understand that all information I give will be confidential and held securely in strictest confidence. | |
| I am happy for the research team to contact my GP about my participation in this study. | |
| This section is to be completed **after** the consultation | Initial |
| After completing the Skype consultation, I am still willing for the video material to be used in the research. | |

Participant's Name (capitals) _ _ _ _ _ _ _ _ _ _ _ _ _ Signed _ _ _ _ _ _ _ _ _ _ _ _ _ _ _ __

Researcher signature _ _ _ _ _ _ _ _ _ _ _ _ _ _ _ _ _ _ _ _ _ _ _ _ _ _ _ Date _ _ _ _ _ _

*Table A1: RIAS clusters and categories illustrated with examples from our data*

| CLUSTER / CATEGORY | CODES | DESCRIPTION |
|---|---|---|
| **SOCIO-EMOTIONAL TALK AND BEHAVIOUR** | | |
| Social behaviour | Personal remarks, social conversation | Greetings (e.g. 'How are you today'?), conversation on a non-medical topic (e.g. weather, sport) |
| | Laughs, jokes | Friendly jokes and laughing |
| | Compliments | Expression of approval of someone outside of the clinic encounter (e.g. another doctor or nurse) |
| | Approval | Expression of approval or gratitude towards what the other person has said or done (e.g. 'Thank you', 'Great, well done') |
| Verbal attentiveness | Agreement | Shows agreement or understanding person has said (e.g. 'Okay', 'Yes') |
| | Back-channel' talk (clinician only) | Indicators of sustained interest, attentive listening ('Right [go on.]. 'Yep' [I'm listening]) |
| | Empathy | Recognising the emotional state of the other person (e.g. 'The pain must be very upsetting for you') |
| | Legitimizing statement | Indicating that the other's emotional situation, actions, or thoughts (e.g. " It is natural to feel that way'). |
| Concerns/ reassurance | Shows concern, | Expression indicating that a condition or event is serious or worrisome (e.g. 'I can't seem to control my blood sugar levels well at all'). |
| | Seeks reassurance | Questions of concern that convey the need or desire to be reassured or encouraged (e.g. Do you think it's serious?') |
| | Gives reassurance/optimism | Statements indicating optimism, encouragement, relief of worry or reassurance (e.g. these readings are so much better than before). |
| | Partnership statement | Statements that convey physician's alliance with patient (e.g. but we can work together to find the best insulin dose for you). |
| | Self-disclosure | Statements that describe the physician's personal experiences in areas that have medical and/or emotional relevance for the patient (e.g. 'I've worked with one other person with this type of condition before'). |
| Negative talk | Disagree | Indication of disapproval, criticism, complaint, rejection of what the other person has said or done ("You shouldn't skip your medication like that") |
| | Criticise | Indication of disapproval, complaint, rejection, coolness, or disbelief directed toward another not involved in the exchange (e.g. a different hospital) |
| **TASK-FOCUSED TALK** | | |
| Asking questions | Medical (closed/open] | Questions about medical condition, including closed (e.g. '*Where is the tumor?) and open (e.g. 'tell me about the pain you have been experiencing').* |
| | Therapeutic regime (closed/open] | Questions about ongoing or future treatment plan, including closed ('When is our next appointment?') and open ('What will the treatment involve?). |
| | Lifestyle (closed/open] | Questions about to lifestyle, diet, employment, includes closed (e.g. 'What time do you have breakfast) and open ('What sort of food should I be eating instead?') |
| | Psychosocial (closed/open] | Questions about to psychosocial concerns or problems and social/family life (e.g. 'Are you stressed about this?) and open (e.g. 'How are you feeling?) |
| | Other (closed/open] | Questions that do not fall into the above categories – closed (e.g. 'What date is it today') and open ('Where do I go to get out of the hospital?). |
| Information giving | Medical | Fact/opinion related medical condition, such as symptoms, |

| | | |
|---|---|---|
| | | diagnosis, prognosis, test results (e.g. You've gained 5 pounds since the last visit). |
| | Therapeutic regime | Fact/opinion relating to ongoing or future treatment plan (e.g. 'We will see you in about 6 months) |
| | Lifestyle | Fact/opinion relating to lifestyle, diet, employment (e.g. I go for a walk every morning) |
| | Psychosocial | Fact/opinion relating to psychosocial concerns and social/family life (e.g.' I was feeling quite down about the whole thing'). |
| | Other | Facts/opinions that do not fall into the above categories (e.g. 'Today's date is the 14th of June') |
| Counselling (clinician only) | Medical/Therapy | Statements to suggest resolution or action to be taken for medical problem, drug regimen, future appointments (e.g. 'Increase your morning insulin by 2 unites.') |
| | Lifestyle/Psychosocial | Statements to suggest resolution or action to be taken for lifestyle, family, activities of daily living ('Try to do more exercise during the day'). |
| Requests | Asks for understanding | Mechanism by which the doctor or patient quickly checks with the other to see if information that was just said has been followed or understood (e.g. 'Okay?' 'Does that make sense?) |
| | Asks opinion | Asking for the patient's opinionor perspective ('Do you think it would be good to keep the next appointment?') |
| | Asks permission | Question that specifically ask for permission to give information or to proceed ('May I examine you?') |
| | Requests service (patient only) | Patient-initiated requests for service or treatment (e.g. 'Could you write a prescription for more testing strips') |
| **PROCESS TALK** | | |
| Direction | Orientation | Telling the other what is about to happen or serve to orient or guide them (e.g. 'I am just checking the diary or you', 'please move you head to the side') |
| | Transition | Sentence fragments that indicate movement to another topic or place-holder (e.g. 'Now let us take a look at you records') |
| Checks understanding | Paraphrase/checks | Mechanism for requesting repetition of the other's previous statement (e.g. I didn't quite get that part?') |
| | Bid for repetition | Requesting repetition of the other's previous statement (e.g. 'Could you sat that again please'). |
| **TECHNOLOGY-RELATED TALK AND BEHAVIOUR** | | |
| Socio-emotional | Any socio-emotional category relating to technology | Includes social behaviour (e.g. laughter in response to a technical issue), Verbal attentiveness (e.g. agreement or understanding of technical issues expressed by the other person), concern/reassurance (e.g. seeking reassurance that they can be heard/seen by the other). |
| Task-focused | Any task-focused category relating to technology | Asking questions (e.g. asking if their video is switched on), Information giving (e.g. the volume is on maximum setting), and counselling (e.g. informing patient to message them on the system if they need to get in touch). |
| Process | Any process category relating to technology | Includes orientation (e.g. statements that indicate what they can see on screen to guide consultation), transition (e.g. placeholders to deal with technical issues), and checks understanding (e.g. asking the person to repeat due to technical problems). |

*Figure A1: Routine for a face-to-face consultation in the Antenatal Diabetes Clinic*

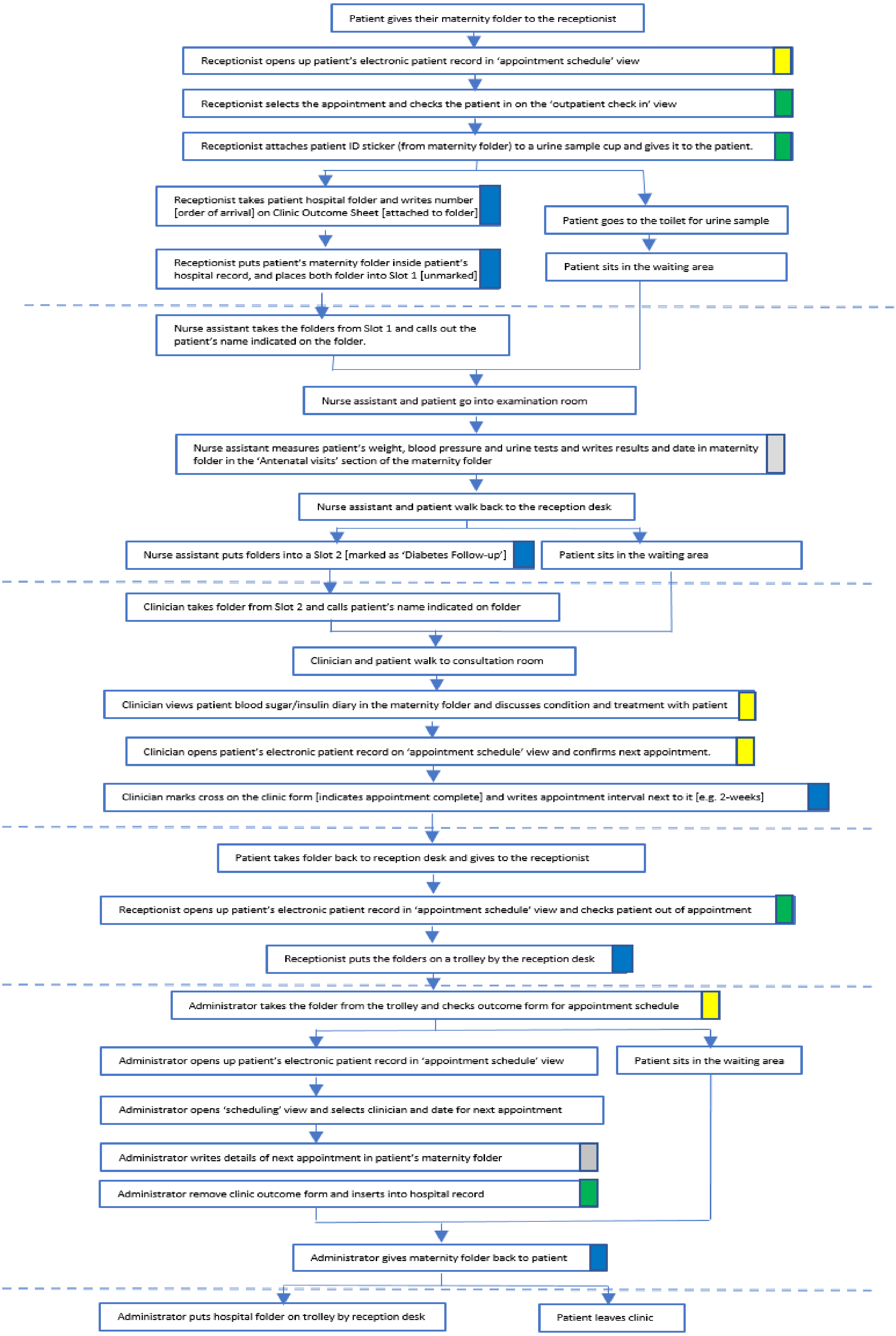

*Figure A2: Routine for a virtual consultation in the Antenatal Diabetes Clinic*

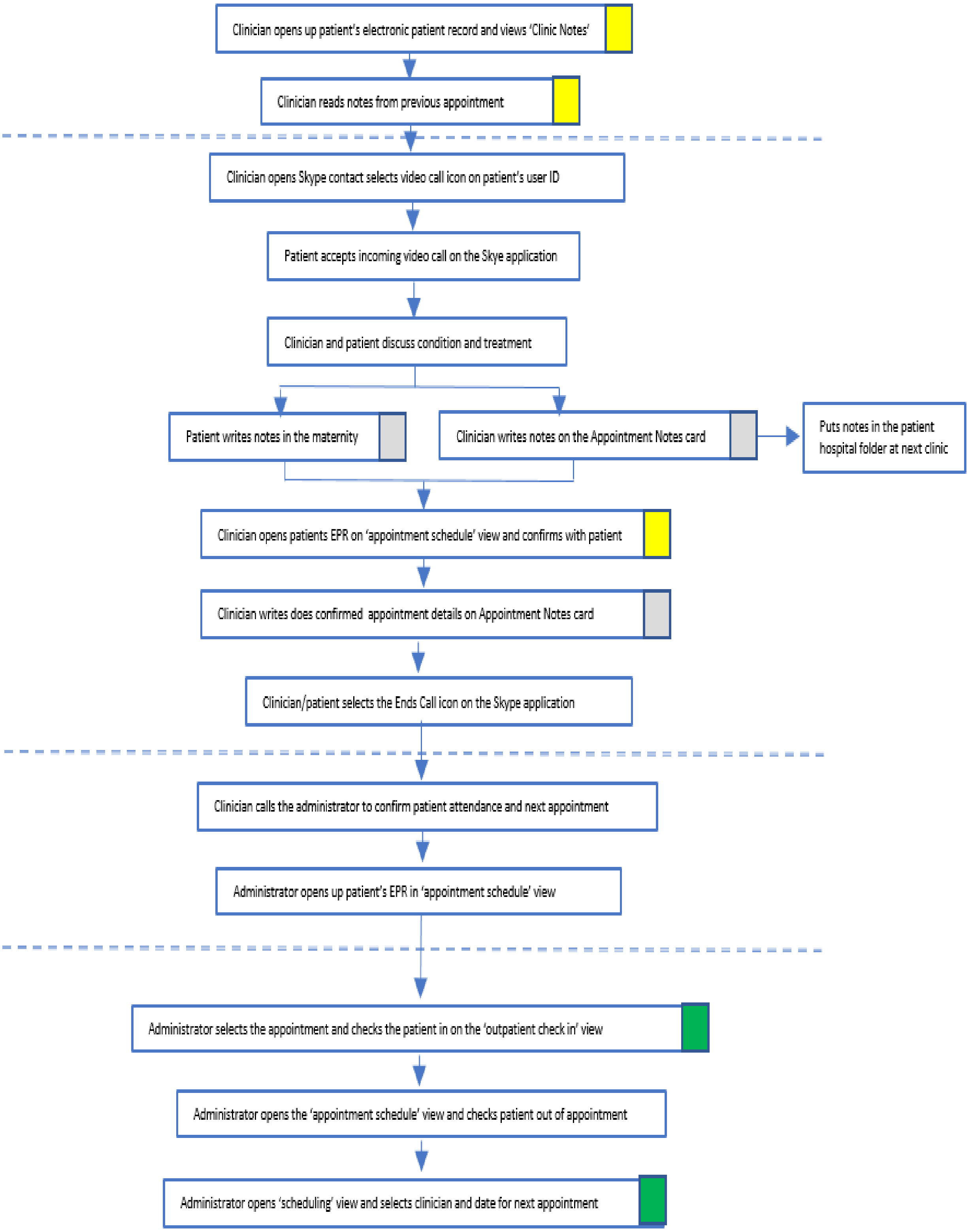


*Figure A3: Routine for a face-to-face consultation following hepatobiliary cancer surgery*

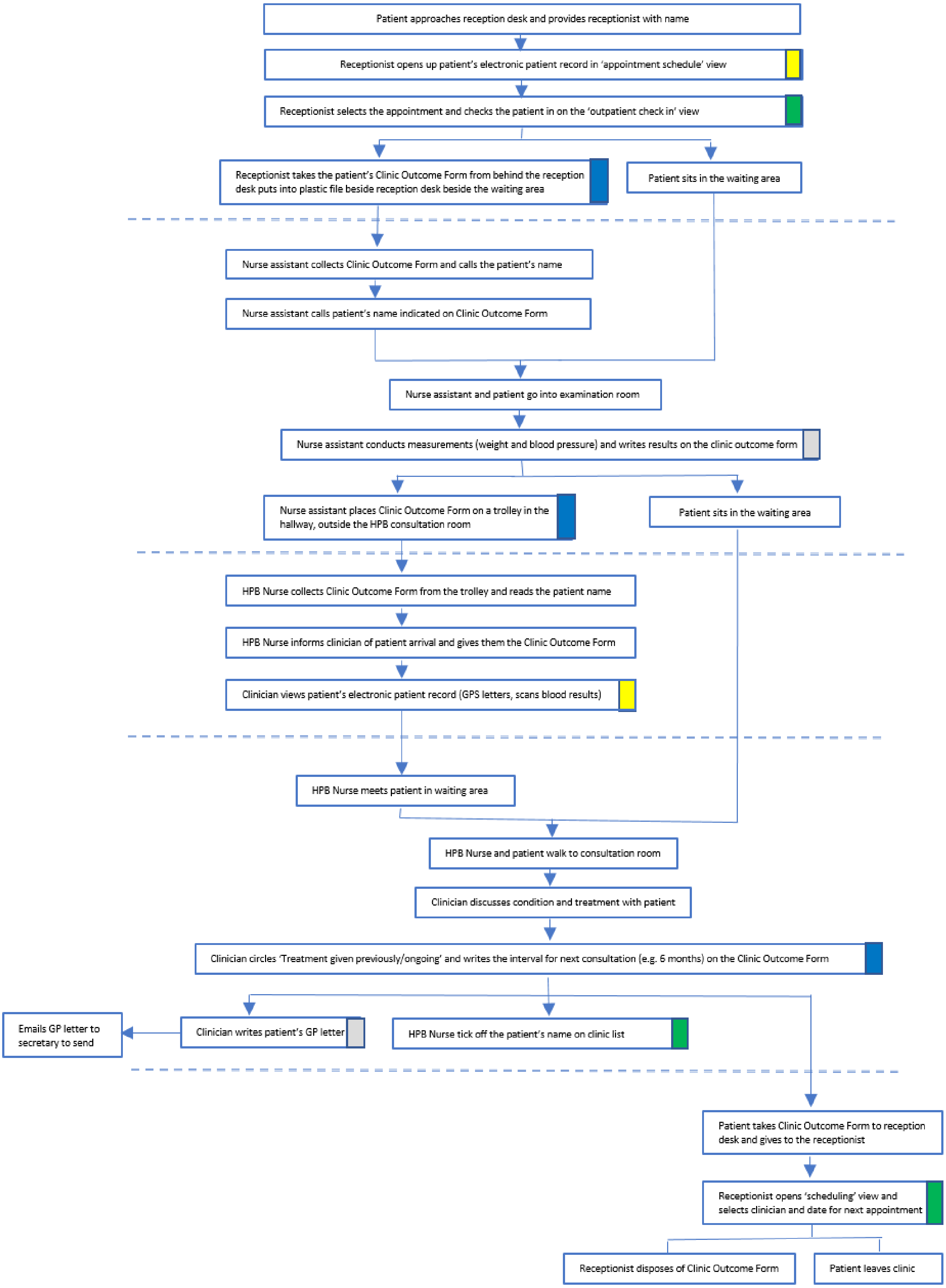


*Figure A4: Routine for a virtual consultation following hepatobiliary cancer*

*surgery*

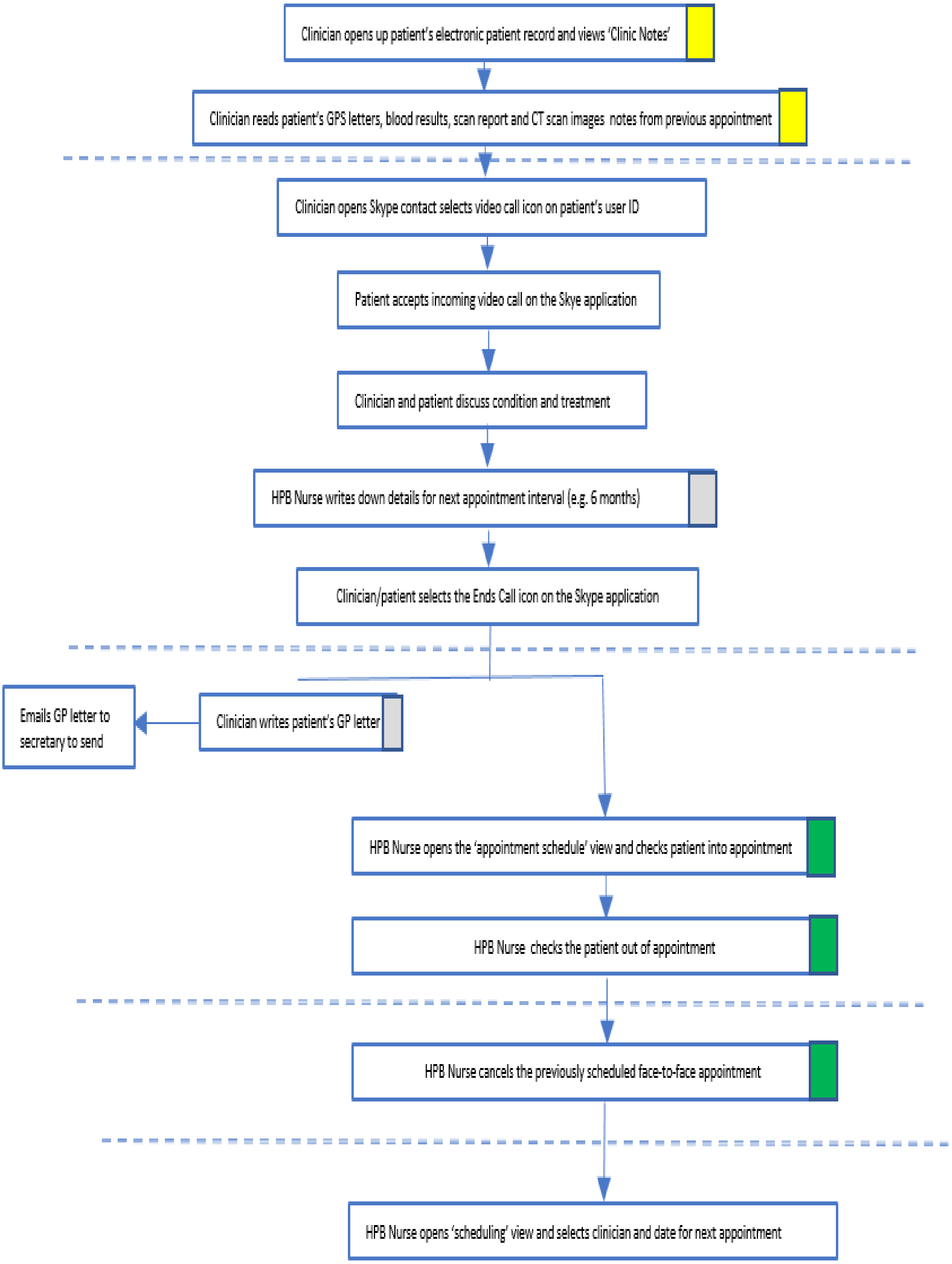

Clinician opens up patient's electronic patient record and views 'Clinic Notes'
Clinician reads patient's GPS letters, blood results, scan report and CT scan images notes from previous appointment
Clinician opens Skype contact selects video call icon on patient's user ID
Patient accepts incoming video call on the Skye application
Clinician and patient discuss condition and treatment
HPB Nurse writes down details for next appointment interval (e.g. 6 months)
Clinician/patient selects the Ends Call icon on the Skype application
Emails GP letter to secretary to send
Clinician writes patient's GP letter
HPB Nurse opens the 'appointment schedule' view and checks patient into appointment
HPB Nurse checks the patient out of appointment
HPB Nurse cancels the previously scheduled face-to-face appointment
HPB Nurse opens 'scheduling' view and selects clinician and date for next appointment
