# Supplementary material for: Real-World Implementation of Video Outpatient Consultations at Macro, Meso, and Micro Levels: Mixed-Method Study
Source: J Med Internet Res. 2018 Apr 17;20(4):e150. doi: 10.2196/jmir.9897 (PMC5930173; doi:10.2196/jmir.9897)
Supplement: Multimedia Appendix 3 [file jmir_v20i4e150_app3.pdf]

VOCAL Project

# Skype Consultations

## Guidance on using Skype and similar virtual media for remote consultations

### About this document

This document has been produced as part of a research collaboration led by Barts Health NHS Trust and Queen Mary University of London. It highlights key lessons on the implementation and use of Skype in a Diabetes service.

### Recommendations for practice

- Introduce the service slowly and incrementally with direct involvement of the team to ensure compatibility between the technology and existing practice.
- .Allow plenty of time for discussion with staff and patients about how it affects the service.
- Work in collaboration with your ICT department and technical support teams to establish roles and processes to assist use of the technology.
- Use with an understanding of the patients' lives and how the technology relates to the management of their health condition.
- Support flexible use, allowing scope to fit the service around the needs of the patient.

## Implementing and Managing Skype

### Installing and running software

The installation of Skype onto NHS computers should be performed by your Trust's IT department. Skype software should be downloaded from the official Skype website (www.skype.com). Skype may require software updates once it has been installed, involving further downloads. It is important to work with your IT department to establish how the downloads and updates can be performed effectively to minimise delay or disruption to the service.

### Managing Skype accounts

*Clinic accounts:* You will need to register your clinic account with a username that is unique to every user and cannot be changed or duplicated once created. Register your Skype username using your Trust NHS email address, as this is more secure than internet based email accounts.

*Patient accounts:* Skype contacts are created by searching other users on the Skype directory and sending a contact invitation.  It is important to ensure that correct contacts are formed during this process. Provide patients with written details of the clinic account username so that they can confirm these before sending or accepting a contact request. Note that some patients will have a username that will not reflect their actual name.

Barts Health NHS Trust | Queen Mary University of London | University of Oxford

The VOCAL Project is funded by the National Institute for Health Research HS&DR Programme
Copyright © 2015 VOCAL project team

# Conducting Skype Consultations

## Communication

Video based communication can help the consultation by providing visual/physical assessment and supporting non-verbal communication. But it is also important to acknowledge potential limitations of video mediated communication and the suitability of this medium depending on the patient and their care support needs. Use Skype adaptively with each patient in conjunction with the use of face to face consultations.

Position the webcam directly above the computer screen in the centre to avoid mismatch between the viewer and camera. This will help create realistic and direct interaction. If you are using other documents or applications during the consultation (e.g. Electronic Patient Record), then display these on the same computer screen when possible to maintain direct interaction.

Patients may send messages for a number of reasons, such as rescheduling appointments, requesting a Skype call or to ask questions about their condition. It is important to make patients aware how regularly you will check messages in order to manage their expectations as to when you will respond.

Ask patients to have other forms of communication available with them at the consultation so that they can be easily contacted if any technical problems occur immediately prior to the Skype call.

## Privacy and Confidentiality

A Skype consultation should be treated like any other outpatient consultation, in which sensitive and confidential information is safeguarded at all times.

Do not conduct the consultation in the presence of others without the patient's permission and take reasonable measures to avoid inadvertent disclosure of information.

Close the office door before initiating or receiving a Skype call and use signs to indicate that a consultation is taking place. Ensure other staff members are aware that Skype is used for consultations within the clinic to minimise risk of unintended interruptions.

Inform patients that Skype cannot protect users from spyware, which can compromise their security. Advise patients to ensure they have adequate anti-virus protection on their computers.

Make patients aware that some personal information from their Skype account is stored locally on the computer. This is particularly important if patients intend to use public or shared computers. Advise patients to logout of their Skype account when not in use.

## Patient Consent

Patients should be made aware of precautions required and potential risks of using Skype. Their consent to use Skype should be documented after they have had time to listen and read the information. The information should ensure that patients are aware and understand the following:

- use of Skype is completely voluntary and they can change a Skype based appointment to a face to face appointment at any time.
- video consultations are encrypted but it is their responsibility to ensure they have adequate anti-spyware and anti-virus protection to prevent unauthorized eavesdropping.
- some personal information is stored locally on the computer being used, which patients should be aware of, particularly if they are using a public or shared computer.
- Skype on a mobile phone is only as secure as any other mobile phone call
- medical outcomes from a consultation will be stored on their patient record
- Skype should not be used in an emergency as a response to calls/messages is not guaranteed

Barts Health NHS Trust
Queen Mary University of London
University of Oxford
The VOCAL Project is funded by the National Institute for Health Research HS&DR Programme
Copyright © 2015 VOCAL project team.

# Standard Operating Procedure - Introducing the Skype Service to Patients

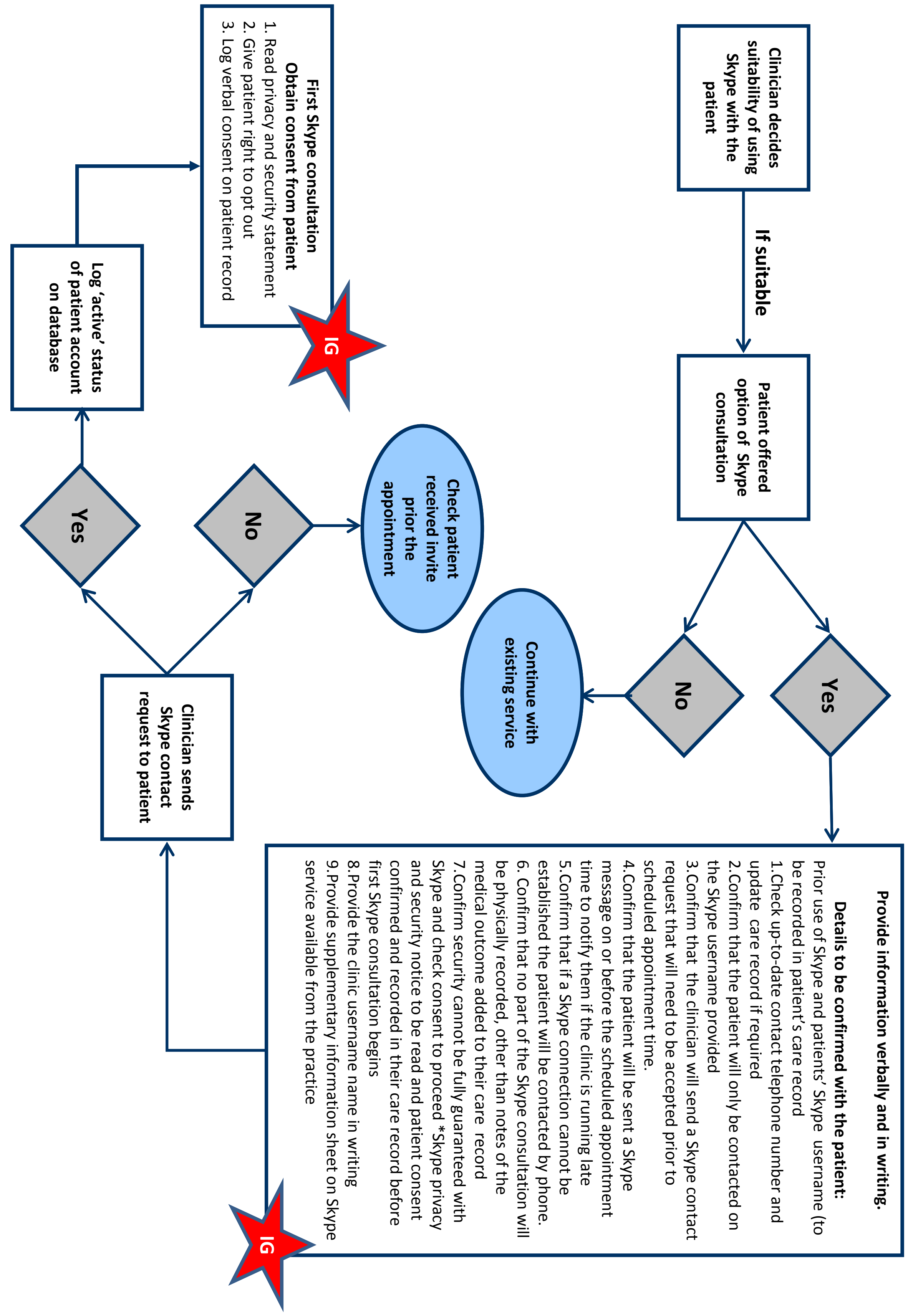


Barts Health NHS Trust

Queen Mary University of London

University of Oxford

Copyright © 2015 VOCAL project team: Contact Joe Wherton (joseph.wherton@phc.ox.ac.uk) if you wish to use this document or require further information
